# Intra-arterial selective hypothermia for acute ischemic stroke neuroprotection: A multicenter pilot trial in China

**DOI:** 10.1371/journal.pmed.1004668

**Published:** 2025-07-24

**Authors:** Zhi-Xin Huang, Miaomiao Hu, Pan Zhang, Xuying He, Xinan Ma, Quanlong Hong, Ping Chen, Huanhuan Luan, Zongyi Wu, Chaolai Liu, Wei Li, Yongkun Li, Yongtao Yu, Min Li, Yajun Li, Dezhi Liu, Zhongkui Han, Weiliang Shi, Jilong Yi, Liang Liu, Guoqiang Xu, Haike Lu, Lizhi Zhou, Xinfeng Liu, Wen Sun, For the ISOLATION Group

**Affiliations:** 1 NeuroMedical Center, The Affiliated Guangdong Second Provincial General Hospital of Jinan University, Guangzhou, Guangdong, China; 2 Department of Neurology, Centre for Leading Medicine and Advanced Technologies of IHM, The First Affiliated Hospital of USTC, Division of Life Sciences and Medicine, University of Science and Technology of China, Hefei, Anhui, China; 3 Department of Neurology, Huaibei Miners General Hospital, Huaibei, Anhui, China; 4 Department of Neurology, Quanzhou First Hospital, Quanzhou, Fujian, China; 5 Department of Neurology, The First Hospital of Putian City, Putian, Fujian, China; 6 Department of Neurology, The Fourth Hospital of Liuan, Liuan, Anhui, China; 7 Department of Neurology, Zhongshan Hospital of Traditional Chinese Medicine, Zhongshan, Guangdong, China; 8 The First People’s Hospital of Jining, Jining, Shandong, China; 9 Department of Neurology, First Affiliated Hospital of Hainan Medical University, Haikou, Hainan, China; 10 Department of Neurology, Shengli Clinical Medical College of Fujian Medical University, Fuzhou, Fujian, China; 11 Department of Neurology, Fuyang Hospital of Anhui Medical University, Fuyang, Anhui, China; 12 Department of Neurology, Jiangsu Province Hospital of Chinese Medicine, Nanjing, Jiangsu, China; 13 Department of Neurology, The First Affiliated Hospital of Anhui University of Traditional Chinese Medicine, Hefei, Anhui, China; 14 Department of Neurology, The First Affiliated Hospital of Nanjing Medical University, Nanjing, Jiangsu, China; 15 Department of Neurology, Fuyang Tumour Hospital, Fuyang, Anhui, China; 16 Department of Neurology, Haiyan People’s Hospital, Jiaxing, Zhejiang, China; 17 Department of Neurology, Central Hospital of Jingmen, Jingmen, Hubei, China; 18 Department of Neurology, Dingyuan General Hospital, Dingyuan, Anhui, China; 19 Department of Neurology, The First People’s Hospital of Yongkang, Yongkang, Zhejiang, China; 20 Department of Biostatistics, School of Public Health, Southern Medical University, Guangzhou, Guangdong, China; Barts and the London School of Medicine & Dentistry Queen Mary University of London, UNITED KINGDOM OF GREAT BRITAIN AND NORTHERN IRELAND

## Abstract

**Background:**

Acute ischemic stroke (AIS) remains a leading cause of disability and death globally, with limited effective neuroprotective strategies beyond reperfusion therapies. Despite advances in reperfusion treatments, many patients still experience poor outcomes, highlighting the urgent need for additional therapeutic approaches. We investigated whether intra-arterial local therapeutic hypothermia (IA-LTH) combined with endovascular treatment could improve outcomes in patients with AIS.

**Methods and findings:**

We conducted a multicenter, randomized trial with blinded outcome assessment (ISOLATION trial), where outcome assessors and patients were blinded to treatment allocation while procedural staff could not be blinded, to test the effectiveness of IA-LTH for neuroprotection in AIS (registration number: ChiCTR2300074990). Between September 2023 and January 2024, we recruited 100 patients with anterior circulation large vessel occlusion within 24 h of stroke onset from 18 stroke centers in China. Participants were randomly assigned (1:1) to receive either IA-LTH plus standard care (including endovascular treatment and guideline-recommended medical therapy; intervention; *n* = 50) or standard care alone (control; *n* = 50). The IA-LTH group received intra-arterial infusion of 4 °C saline during and following endovascular treatment. Primary outcome was favorable functional outcome (modified Rankin Scale 0–2) at 90 days. Using intention-to-treat analysis, among participants (median age 69 years, 64% [64/100] male, median National Institutes of Health Stroke Scale score 14), the primary outcome did not reach statistical significance, with favorable outcome achieved in 58.0% (29/50) of the IA-LTH group versus 40.0% (20/50) of controls (adjusted relative risk [aRR] of 1.47 (95% CI [0.99, 2.16]; *P* = 0.055)). This lack of statistical significance is primarily due to the limited sample size of this pilot study, which was designed to assess feasibility and safety rather than provide definitive efficacy evidence. Safety outcomes, including rates of symptomatic intracranial hemorrhage (8.0% [4/50] versus 16.0% [8/50]) and mortality (20.0% [10/50] versus 22.0% [11/50]), were not significantly different between groups. The main limitations include the insufficient sample size of this pilot study which limited statistical power to detect differences in the primary outcome, the inability to adjust for potentially important confounders beyond age and ASPECTS due to the small sample size, and the higher incidence of pulmonary infections in the IA-LTH group that may have resulted from hypothermia-induced immune suppression or sedation-related factors.

**Conclusions:**

This pilot study provides preliminary insights suggesting that IA-LTH combined with endovascular therapy may be feasible and safe, with potential to improve functional outcomes in patients with AIS. The primary outcome did not reach statistical significance. However, the observed numerical differences suggest that IA-LTH warrants further investigation in larger trials.

**Trial registration:**

Chinese Clinical Trial Registry (ChiCTR) ChiCTR2300074990

## Author summary

**Why was this study done?:**

- Acute ischemic stroke remains a leading cause of disability and death despite advances in treatment
- Therapeutic hypothermia has shown neuroprotective potential, but systemic cooling faces implementation challenges
- Local brain cooling through endovascular approach could provide targeted neuroprotection with fewer complications

**What did the researchers do and find?:**

- We conducted a multicenter randomized trial with 100 patients who had acute ischemic stroke, comparing intra-arterial cold saline infusion during endovascular treatment to standard care alone
- For our primary outcome, favorable functional status at 90 days was achieved in 58.0% of IA-LTH patients versus 40.0% of controls, though this difference did not reach statistical significance
- Safety outcomes showed favorable numerical values with lower rates of symptomatic hemorrhage and mortality in the IA-LTH group

**What do these findings mean?:**

- This study demonstrates that targeted brain cooling through endovascular approach is feasible and could be further investigated as a treatment option
- The intervention’s favorable safety profile supports its further investigation in larger trials
- The main limitations were the small number of patients in this early-stage trial which made it difficult to detect certain differences between treatments, the inability to keep medical staff unaware of which treatment patients received due to the nature of the cooling procedure, and the higher occurrence of chest infections in the cooling group which needs to be monitored in future studies

## Abstract 3

In a multicenter pilot trial, Zhi-Xin Huang and colleagues investigate if intra-arterial local therapeutic hypothermia combined with endovascular treatment could improve outcomes in patients with acute ischemic stroke.

## Introduction

An estimated 7.6 million people have an acute ischemic stroke (AIS) each year, making it one of the leading causes of death and disability worldwide [1]. Despite significant advancements in reperfusion approaches such as endovascular therapy (EVT) and intravenous thrombolysis, many patients still do not achieve functional independence post-treatment [2–4]. This reality highlights the urgent need for more effective treatment methods, especially in the field of neuroprotection.

In recent years, EVT has been shown to significantly improve the prognosis at 90 days in specific patient populations, yet even so, only 46%–60% of patients achieve functional independence [5,6]. In China, the standard of care for patients with AIS follows national guidelines similar to international recommendations, including intravenous thrombolysis for eligible patients within 4.5 h after symptom onset and endovascular thrombectomy for those with large vessel occlusion within 24 h, with ongoing efforts to improve implementation across diverse healthcare settings nationwide. Moreover, the therapeutic window for reperfusion treatment has been extended from 8 to 24 h, providing more patients with treatment opportunities [7]. However, reperfusion itself can trigger a series of pathological processes, including astrocyte swelling, pericyte contraction, and platelet aggregation, ultimately leading to microcirculatory failure, known as the “no-reflow” phenomenon [8–10].

In response to these challenges, neuroprotective strategies have emerged to reduce ischemic brain damage and enhance the effectiveness of reperfusion therapies. Therapeutic hypothermia, as a multifaceted neuroprotective approach, has shown significant effects in cardiac arrest and neonatal hypoxic-ischemic encephalopathy [11–13]. However, the application of hypothermia in the treatment of AIS has not yet been widely adopted, partly due to the systemic complications associated with whole-body hypothermia [14].

Despite previous research exploring various hypothermia neuroprotection techniques, including local surface cooling, intranasal devices, and endovascular cooling catheters, these methods have not been ideal in clinical practice, mainly because they may not effectively avoid the systemic complications associated with whole-body hypothermia [15,16]. These complications include shivering, bradycardia, hypertension, etc., which may negate the potential benefits of hypothermia treatment.

In light of these challenges, this study adopts an approach using intra-arteria local therapeutic hypothermia (IA-LTH) treatment, aiming to achieve rapid, selective brain hypothermia induction while minimizing systemic complications. The key to this method is the precise control of the delivery of cooling fluids, directly targeting the ischemic area, thereby protecting local brain tissue without reducing overall body temperature.

Recent non-randomized studies suggest potential benefits of IA-LTH in reducing infarct volume post-stroke [17], yet its impact on neurological outcomes remains unclear. To address this gap, this pilot study aims to evaluate the efficacy and safety of IA-LTH in patients undergoing mechanical thrombectomy for acute cerebral infarction. We hypothesize that combining IA-LTH with mechanical thrombectomy may improve functional outcomes by mitigating reperfusion injury.

As a pilot study, our primary objective is to establish preliminary safety and efficacy data, providing a foundation for future large-scale trials. Specifically, we aimed to test the hypothesis that combining IA-LTH with mechanical thrombectomy would improve functional outcomes at 90 days compared to standard care alone in patients with anterior circulation large vessel occlusion. This research addresses gaps in current literature and may contribute to understanding potential treatment approaches for AIS. The findings may have significant implications for optimizing treatment protocols in this critical field of medicine.

## Methods

### Trial design

This study was conducted as a multicenter, prospective, open-label, randomized, blinded-endpoint trial across 18 centers in China. The protocol (S1–S3 Appendix), available with the full text of this article at supplemental materials, was approved by the Ethics Committee of Guangdong Second Provincial General Hospital (approval number: 2023-KY-KZ-100-03) as the central institutional review board. A separate, impartial review board supervised trial safety and data quality, and an independent clinical endpoint adjudication committee was responsible for evaluating any adverse events. The aim of this study was to assess the safety, feasibility, and potential efficacy of IA-LTH combined with reperfusion therapy in patients with AIS within 24 h of symptom onset. All study participants or their legal proxies provided documented informed consent prior to enrollment. The trial adhered to Declaration of Helsinki principles and International Council for Harmonization Good Clinical Practice guidelines. The authors vouch for protocol fidelity and data integrity. This study is reported in accordance with the Consolidated Standards of Reporting Trials (CONSORT) 2025 guideline for randomized trials (see S1 CONSORT Checklist). Additionally, we have completed the SPIRIT 2025 checklist for our trial protocol (see S1 SPIRIT Checklist).

### Participants

Subjects were adults aged 18–85 years who presented with AIS, independently functioning before stroke (Modified Rankin Scale [mRS] evaluations of 0–1; scoring spectrum from 0 [asymptomatic] to 6 [mortality]), enrolled within 24 h of symptom onset (defined as the time the patient was last seen well). Imaging (computed tomography angiography [CTA], magnetic resonance angiography [MRA], or digital subtraction angiography) confirmed anterior circulation large vessel occlusion (internal carotid artery or M1 segment of the middle cerebral artery).

Key exclusion criteria were: pre-existing mRS score >1; acute stroke lesions in both cerebral hemispheres and/or anterior and posterior circulations simultaneously; CT or MRI confirming cerebral hemorrhage/subarachnoid hemorrhage; presence of active bleeding, severe anemia, or coagulation disorders; cardiac function worse than Class I, with history of severe failure or high risk of acute decompensation or fluid intolerance; severe cardiac, hepatic, or renal diseases; concomitant malignancies or other diseases with a life expectancy less than 3 months; and current participation in other clinical trials.

Patients receiving intravenous thrombolysis or EVT were eligible. Protocol required assessment of operative feasibility. Patients or guardians needed to comprehend the trial and commit to follow-up. Full eligibility criteria are in the supplementary protocol.

### Randomization and blinding

This study utilized one-to-one stratified block randomization. Stratification factors included age (≥75 years and ≥18 to <75 years) and Alberta Stroke Program Early CT Score (ASPECTS) (≥8 and ≤7). Randomization was implemented through a central Interactive Web Response System using a SAS-generated randomization list. The study employed competitive enrollment across centers. To maintain allocation concealment, an independent statistician prepared the randomization list. While complete blinding was unfeasible due to the intervention’s nature, several measures were implemented to minimize bias, such as blinded evaluation procedure was set up for the primary outcome evaluation (90-day mRS), and a blinded central adjudication committee was set up to review all outcomes and safety events. Additionally, personnel involved in the study and patients were instructed not to disclose treatment information to outcome assessors. These procedures aimed to ensure study validity and minimize potential biases in treatment allocation and outcome assessment.

### Procedures

Randomization occurred at the start of cerebral vascular intervention. Thrombectomy using combined contact aspiration and stent retriever was recommended for all patients. In the treatment group, after successful microcatheter placement beyond the occlusion, 50 ml of 4 °C saline was infused at 10 ml/min, followed by standard thrombectomy. Regardless of reperfusion success, an additional 300 ml of 4 °C saline was infused at 30 ml/min through the intermediate or guiding catheter in the internal carotid artery. Room temperature contrast was generally avoided during angiography to maintain hypothermia. If reperfusion failed, a second hypothermic treatment was administered. When microcatheter could not go through the site of occlusion or direct aspiration was employed without the use of microcatheter, 350 ml of 4 °C saline at 30 ml/min was administered post-procedurals. The control group underwent identical procedures with room temperature saline. To ensure consistency and reduce operational variability across centers, researchers received training on the trial protocol, including efficacy and safety assessment criteria, as well as standardized surgical and hypothermic techniques. Detailed procedures are available in the S2 Appendix.

National Institutes of Health Stroke Scale (NIHSS) assessments were conducted at baseline, 1 day, and 7 days post-randomization. Demographic and clinical data were collected at randomization, with follow-ups at 1 day, 7 days (or discharge if earlier), and 90 days. The study maintained stringent quality control measures, including remote and on-site monitoring and data verification, to ensure procedural fidelity and accuracy. All clinical and imaging data were centrally analyzed for consistency. Participants received standard medical care following national stroke guidelines.

### Outcomes

The primary outcome was the attainment of a favorable functional status, defined as a score of 0–2 on mRS at 90 days (±7 days) post-treatment. Secondary outcomes included the ordinal distribution of mRS scores at 90 days (±7 days), the proportion of patients achieving excellent functional status (mRS score of 0–1 at 90 days [±7 days]), and the incidence of early neurological deterioration (END), defined as an increase of 4 or more points on the NIHSS within 24 hthat was not attributable to intracerebral hemorrhage. Secondary outcomes included reperfusion success rate (defined as modified Treatment in Cerebral Infarction score ≥ 2b) assessed immediately post-procedure, infarct volume on CT or MRI obtained between 24 h and 7 days post-procedure, recurrent occlusion rate on MRA or CTA performed between 24 h and 7 days post-procedure, and change in rectal temperature immediately before and after treatment. The study also analyzed all-cause mortality within 90 days post-randomization as an additional post-hoc outcome, which was implicitly measured as part of the mRS assessment (score of 6 representing death) and safety monitoring.

Predefined safety outcomes encompassed any adverse or serious adverse events (SAEs) during IA-LTH, such as pulmonary infections, symptomatic intracranial hemorrhage (sICH) defined as ICH associated with a worsening of 4 or more points on the NIHSS or resulting in death. We also monitored cerebral herniation as an additional safety outcome, although this was not pre-specified in the original protocol. Additionally, the safety profile assessment included monitoring for coagulation abnormalities and bradycardia, recognizing the potential impact of hypothermia on hemostasis and cardiac function. Adverse events were adjudicated by the data safety monitoring board chairman. Brain hemorrhage was assessed via CT or MRI performed 24–36 h post-procedure (falling within the protocol-specified window of 72 h) without the use of intravenous contrast agents. The 90-day mRS scores were evaluated either through in-person visits or telephone interviews. Assessment and documentation of adverse events adhered to the National Cancer Institute’s Common Terminology Criteria for Adverse Events (CTCAE) version 5.0 guidelines.

### Statistical analysis

For this pilot study, the sample size of 100 cases was determined based on recommendations from the Steering Committee without a formal calculation, as stated in our Statistical Analysis Plan. This approach is consistent with previous pilot trials of therapeutic hypothermia in AIS, such as the study by Chen and colleagues [17] (*n* = 26) and work by Wan and colleagues [18] (*n* = 142). This sample size was deemed sufficient to assess the feasibility and safety of the intervention while providing preliminary data to inform the design of future larger-scale trials, following methodological guidance for pilot studies [19]. The statistical analysis plan, which is available in S4 Appendix, guided our approach to data analysis. We employed an intention-to-treat (ITT) approach, including all randomized patients except those lost to follow-up or who withdrew consent. Baseline characteristics and procedural details were compared using appropriate statistical tests. Continuous variables were analyzed with Student *t* test or Mann–Whitney *U* test, depending on data distribution. Categorical variables were assessed using chi-squared or Fisher’s exact tests as appropriate.

The primary treatment effect was expressed as a relative risk (RR) with 95% confidence interval (CI), calculated through log-binomial regression. Ordinal logistic regression was used for shift analysis of 90-day mRS scores. Primary outcomes were adjusted for prespecified stratification factors, including age and ASPECTS. Adverse events are reported as RRs with corresponding 95% CI.

All data analyses were conducted with IBM SPSS Statistics version 26 (IBM Corp., Armonk, NY, USA), adopting a two-tailed significance level (*P*) of 0.05. This approach aimed to provide robust and unbiased estimates of treatment effects while accounting for potential sources of variation.

This Intra-arteria local therapeutic hypothermia in patientS with acute anteriOr circulation LArge vessel occlusion Treated with endovascular reperfusION (ISOLATION) trial was registered with the Chinese Clinical Trial Registry (www.chictr.org.cn) under the registration number ChiCTR2300074990 on August 22, 2023, and has now been closed at all participating sites.

## Results

### Trial population

**Fig 1:**
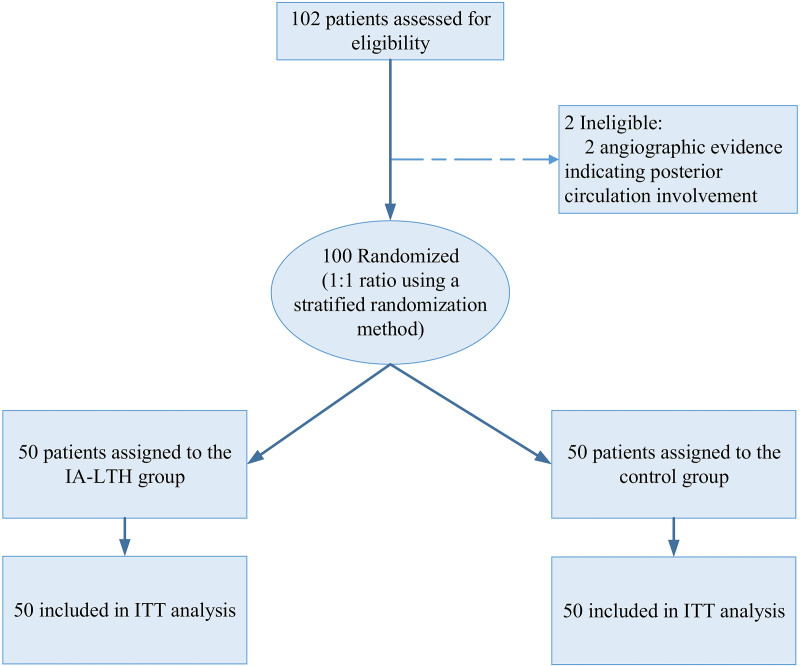
CONSORT flow diagram of patient screening, randomization, and analysis. All randomized patients (*n* = 100) were included in the intention-to-treat (ITT) analysis.

Between September 24, 2023, and January 10, 2024, 102 individuals were screened for preliminary evaluation. Two participants were subsequently excluded due to angiographic evidence indicating posterior circulation involvement. This exclusion was based on predefined criteria to ensure the homogeneity of the study population. After thorough screening, 100 participants met the inclusion criteria and were systematically allocated to either the IA-LTH group (*n* = 50) or the control group (*n* = 50). Ultimately, the ISOLATION study population comprised 100 patients, evenly distributed between the two groups, forming the basis for the analysis. Notably, all 100 patients completed the study procedures as stipulated in the protocol, with no recorded deviations (Fig 1).

Baseline characteristics were well-balanced between the two groups and largely representative of the expected patient population (Table 1). This balance was evident across demographic, clinical, time-based metrics, complications, and imaging characteristics. Of the 100 patients enrolled, the median age was 69 years (interquartile range [IQR], 59–78 years), with 64/100 (64.0%) being male. The median baseline NIHSS score was 14 (IQR, 10–17), reflecting moderate to severe stroke severity. The median time interval from last known well to successful arterial sheath placement was 5 h (IQR, 4–7 h). Notably, 31% (31/100) of patients received intravenous thrombolysis treatment.

**Table 1:** Baseline characteristics of selective endovascular hypothermia and control groups.

Dependent: Selective Endovascular Hypothermia		Control (*n* = 50)	IA-LTH (*n* = 50)
**Demographic Characteristics**			
Age, years	Mean ± SD	69.5 ± 10.9	67.1 ± 12.1
Age, years			
<75	No. (%)	30 (60%)	32 (64%)
≥75	No. (%)	20 (40%)	18 (36%)
Sex (Female)	No. (%)	16 (32%)	20 (40%)
**Health Conditions**			
Hpertension	No. (%)	30 (60%)	27 (54%)
Diabetes	No. (%)	7 (14%)	13 (26%)
Dyslipidemia	No. (%)	2 (4%)	2 (4%)
Coronary Heart Disease	No. (%)	7 (14%)	6 (12%)
Stroke	No. (%)	6 (12%)	10 (20%)
Atrial Fibrillation	No. (%)	13 (26%)	12 (24%)
Heart Valve Disease	No. (%)	3 (6%)	3 (6%)
**Lifestyle Habits**	No. (%)		
Smoking	No. (%)	12 (24%)	9 (18%)
Drinking	No. (%)	13 (26%)	10 (20%)
**Treatment Status**	No. (%)		
Antiplatelet	No. (%)	7 (14%)	8 (16%)
Anticoagulation	No. (%)	5 (10%)	2 (4%)
Statin	No. (%)	8 (16%)	4 (8%)
**Clinical Assessment**			
Aspects	Mean ± SD	7.8 ± 1.6	7.4 ± 1.9
Aspects			
<8		18 (36%)	20 (40%)
≥8		32 (64%)	30 (60%)
NIHSS	Mean ± SD	13.9 ± 4.9	14.4 ± 5.8
mRS before stroke			
0	No. (%)	47 (94%)	44 (88%)
1	No. (%)	3 (6%)	6 (12%)
**Therapeutic Approach**			
Intravenous Thrombolysis	No. (%)	13 (26%)	18 (36%)
**Time-based Metrics**			
TSSP, hours	Median (IQR)	5.0 (3.7 to 6.0)	5.1 (4.2 to 8.3)
TSCTH, hours	Median (IQR)	6.5 (5.4–7.7)	6.5 (5.2–9.4)
TORC, hours	Median (IQR)	6.2 (5.2–7.7)	6.2 (5.1–9.4)
TSEET, hours	Median (IQR)	6.7 (5.7–8.3)	6.8 (5.6–9.7)

**Abbreviations:** IA-LTH, intra-arterial local therapeutic hypothermia; mRS, modiﬁed Rankin Scale; No. (%): Number (percentage); SD, standard deviation; TSSP, Time from symptom onset to successful sheath placement; TSCTH, Time from symptom onset to the completion of therapeutic hypothermia; TSEET, Time from symptom onset to the end of endovascular treatment; TORC, Time from symptom onset to reperfusion completion.

### Primary and secondary outcomes

**Fig 2:**
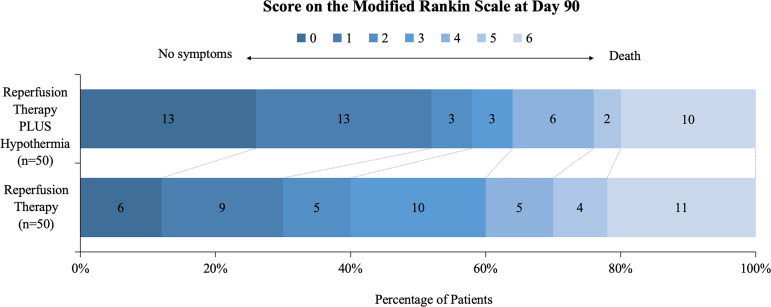
90-Day Modified Rankin Scale (mRS) score distribution. This figure depicts the 90-day mRS score distribution among two patient groups: those treated with IA-LTH (*n* = 50) and those with Reperfusion Therapy alone (*n* = 50). Scores are displayed on a scale from 0 (no symptoms) to 6 (death), with horizontal bars indicating the percentage of patients at each score level and numbers within each segment showing the absolute count of patients. Darker bars signify better outcomes (lower mRS scores).

**Fig 3:**
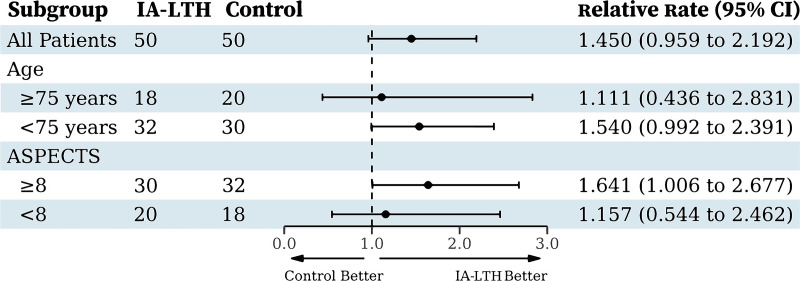
Relative risk of 90-day mRS 0-2 across prespecified subgroups. This forest plot displays the relative risks (RR) and 95% confidence intervals (CI) for the primary outcome of achieving a modified Rankin Scale score of 0 (no symptoms) or 2 (slight disability) at 90 days, comparing the IA-LTH (intervention) group to the control group across various prespecified patient subgroups.

For the primary outcome, the proportion of mRS score 0–2 at 90 days was 58.0% (29/50) in the IA-LTH group and 40.0% (20/50) in the control group. The adjusted relative risk (aRR) was 1.47 (95% CI [0.99, 2.16]), with the result did not achieve statistical significance (*P* = 0.055; Table 2 and Fig 2). To further explore the treatment effect, subgroup analysis was conducted for the primary outcome (Fig 3). The primary outcome of an mRS score of 0 or 2 at 90 days was evaluated across these prespecified subgroups. The overall RR for all patients was 1.45 (95% CI [0.96, 2.19]; *P* = 0.078), indicating a 45% higher likelihood of achieving the favorable outcome with the IA-LTH intervention compared to control, though this difference was not statistically significant. In the subgroup analysis, patients with ASPECTS ≥8 showed a statistically significant RR of 1.64 (95% CI [1.01, 2.68]), suggesting a 64% increased chance of a favorable mRS score with IA-LTH. However, the treatment effect was less clear in other subgroups. For patients aged ≥75 years (RR 1.11 (95% CI [0.44, 2.83])) and those aged <75 years (RR 1.54 (95% CI [0.99, 2.39])), as well as for those with ASPECTS <8 (RR 1.16 (95% CI [0.54, 2.46])), the results were not statistically significant.

**Table 2:** Selective endovascular hypothermia: analysis of primary and secondary outcomes.

	IA-LTH group (*n* = 50)	Control group (*n* = 50)	Unadjusted		Adjusted^a^	
			Effect Size (95% CI)	*P*	Effect Size (95% CI)	*P*
**Primary outcome**						
mRS score 0–2 at 90 days^b^	29/50 (58.0%)	20/50 (40.0%)	1.45 (0.96, 2.19)	0.078	1.47 (0.99, 2.16)	0.055
**Secondary outcomes**						
mRS score 0–1 at 90 days^b^	26/50 (52.0%)	15/50 (30.0%)	1.73 (1.05, 2.86)	0.031^c^	1.76 (1.09, 2.84)	0.020^c^
Improvement in mRS according to category at day 90^d^			1.80 (0.89, 3.63)	0.100	1.78 (0.88, 3.59)	0.109
0	13/50 (26.0%)	6/50 (12.0%)				
1	13/50 (26.0%)	9/50 (18.0%)				
2	3/50 (6.0%)	5/50 (10.0%)				
3	3/50 (6.0%)	10/50 (20.0%)				
4	6/50 (12.0%)	5/50 (10.0%)				
5	2/50 (4.0%)	4/50 (8.0%)				
6	10/50 (20.0%)	11/50 (22.0%)				
Reperfusion success rate^b^	42/50 (84.0%)	39/50 (78.0%)	1.08 (0.89, 1.30)	0.446	1.14 (0.95, 1.37)	0.149
Infarct volume, Median (IQR)^e^	56.5 (52.3–60.9)	57.8 (56.3–64.0)	0.03 (<0.001, 7.84)	0.217	0.03 (<0.001, 7.88)	0.218
Recurrent occlusion rate^b^	2/50 (4.0%)	3/50 (6.0%)	0.67 (0.12, 3.82)	0.649	0.72 (0.13, 3.95)	0.700
∆rectal temperature^f^, mean (SD)^e^	0.5 (0.2)	0.3 (0.2)	1.16 (1.01, 1.25)	<0.001^c^	1.16 (1.08, 1.24)	<0.001^c^
Early neurological deterioration within 24 h after EVT^g^^,^^b^	8/50 (16.0%)	8/50 (16.0%)	1.00 (0.41, 2.46)	1.000	1.02 (0.41, 2.50)	0.974
Death within 90 days	10/50 (20.0%)	11/50 (22.0%)	0.91 (0.43, 1.95)	0.806	0.98 (0.46, 2.06)	0.948

^a^Prespecified grouping variables were adjusted (age, ASPECTS).

^b^Statistical data are shown as percentages. The impact of intervention is displayed using relative risks (with 95% confidence intervals) comparing HDP participants to controls, examined through unmodified and modified log-binomial logistic models. Treatment outcomes are expressed as geometric mean proportions. Every analysis utilized bilateral testing. No corrections were applied for repeated comparisons.

^c^*P* < 0.05.

^d^A shift analysis of ordinal data was used to assess scores across all seven levels of the mRS (ranging from 0 [no symptoms] to 6 [death]).

^e^A generalized linear model was used to assess rectal temperature changes before and after treatment, as well as post-operative infarct volumes (with temperature measured in °C and infarct volumes in mL).

^f^Perioperative rectal temperature changes.

^g^NIHSS score increase of four within 24 h was defined as early neurological deterioration, but not cerebral hemorrhage.

**Abbreviations:** IA-LTH, intra-arterial local therapeutic hypothermia; SD, standard deviation; mRS; modiﬁed Rankin scale.

Secondary outcomes are also presented in Table 2. The proportion of mRS score 0–1 at 90 days was 52.0% (26/50) in the IA-LTH group and 30.0% (15/50) in the control group, demonstrating a statistically significant difference between the two groups (unadjusted RR 1.73 (95% CI [1.05, 2.86])). After adjustment for prespecified stratification factors, the RR remained significant, with an aRR of 1.76 (95% CI [1.09, 2.84]; *P* = 0.020). Furthermore, in the subgroup of patients with ASPECTS ≥8, the IA-LTH group demonstrated statistically significant improvements in both the primary outcome (20/62 versus 13/62, OR 2.92 (95% CI [1.04, 8.24])) and secondary outcomes (18/62 versus 11/62, OR 2.86 (95% CI [1.02, 8.04])), suggesting that IA-LTH may provide greater benefits in patients with smaller infarct volumes. Additionally, in our study, among patients who did not achieve favorable outcomes (mRS 0–2), the rate of EVT-related vascular reocclusion was 9.8% (5/51). Similarly, among those who did not achieve excellent outcomes (mRS 0–1), the vascular reocclusion rate was 8.5% (5/59), suggesting that reocclusion may play an important role in determining patient prognosis.

Using 90-day ordinal shift analysis, the two groups were not significantly different (adjusted odds ratio 1.78 (95% CI [0.88, 3.59])). No significant difference was found in NIHSS score variation from initial assessment to 24 h post-enrollment (END) across both groups (aRR 1.02 (95%CI [0.41, 2.50])). Of the 100 patients, 21 (21.0%) died during the follow-up period, including 20.0% (10/50) in the IA-LTH group and 22.0% (11/50) in the control group (aRR 0.98 (95% CI [0.46, 2.06]))

### Safety

Adverse events are presented in Table 3. Neither group had significantly different incidences of adverse events or SAEs. sICH occurred in four patients (8.0%) in the IA-LTH group and eight patients (16.0%) in the control group (RR, 0.50 (95% CI [0.16, 1.56])). Cerebral hernia occurred in six patients (12.0%) in the IA-LTH group and five patients (10.0%) in the control group (RR, 1.20 (95% CI [0.39 to 3.68])). The incidence of pulmonary infections was notably high in both groups: 48.0% (24/50) in the IA-LTH group and 40.0% (20/50) in the control group. While the RR of pulmonary infections in the IA-LTH group compared to the control group was 1.20 (95% CI [0.77, 1.87]), indicating no statistically significant difference between groups, the overall high rate of this complication warrants attention in the clinical management of patients with acute stroke undergoing endovascular procedures. No cases of rebound fever were also observed in either the IA-LTH or control group during post-procedure monitoring.

**Table 3:** Surveillance and documentation of adverse events and safety endpoints.

	IA-LTH group (*n* = 50)	Control group (*n* = 50)	*Effect Size (95% Confidence Interval)* ^a^
Symptomatic intracranial hemorrhage	4/50 (8%)	8/50 (16%)	0.50 (0.16, 1.56)
Pulmonary infection	24/50 (48%)	20/50 (40%)	1.20 (0.77, 1.87)
Cerebral hernia	6/50 (12%)	5/50 (10%)	1.20 (0.39, 3.68)
Coagulation abnormalities	1/50 (2%)	1/50 (2%)	1.00 (0.06, 15.55)
Bradycardia^b^	0/50 (0%)	3/50 (6%)	
Death during hospitalization	7/50 (14%)	7/50 (14%)	1.00 (0.38, 2.64)

^a^The analyses were not adjusted for multiplicity.

^b^*P* = 0.998.

**Abbreviations:** IA-LTH, intra-arterial local therapeutic hypothermia.

## Discussion

In this investigator-initiated, randomized, multicenter trial, we evaluated the efficacy and safety of IA-LTH as an adjunct to reperfusion therapy in patients with AIS. Our results revealed a numerical difference suggesting a potential benefit of IA-LTH for the primary outcome of achieving mRS scores of 0–2 at 90 days (58.0% versus 40.0%). However, this difference did not reach statistical significance (aRR 1.47 (95% CI [0.99, 2.16], *P* = 0.055)). Notably, the IA-LTH group demonstrated a significantly higher proportion of patients achieving excellent functional outcomes (mRS 0–1) at 90 days compared to the control group (52.0% versus 30.0%, *P* = 0.031), indicating enhanced neurological recovery. The distinction between favorable outcomes (mRS 0–2) and excellent outcomes (mRS 0–1) may suggest that IA-LTH is particularly beneficial for patients capable of achieving near-complete recovery. Additionally, in the subgroup of patients with ASPECTS ≥8, IA-LTH was associated with significant differences in both primary and secondary outcomes, potentially highlighting a subgroup with smaller infarct volumes that is more likely to benefit from this intervention. EVT-related vascular reocclusion may also be a critical determinant of patient prognosis. In our study, among patients who did not achieve favorable outcomes (mRS 0–2), the reocclusion rate was 9.8% (5/51), while in patients who did not achieve excellent outcomes (mRS 0–1), it was 8.5% (5/59). This finding reinforces existing evidence that vascular reocclusion after EVT is a key factor influencing outcomes [20]. Future studies should further investigate the relationship between reocclusion and clinical outcomes to inform targeted interventions.

Safety profiles showed mixed results between groups. While the IA-LTH group demonstrated a lower rate of sICH (8.0% [4/50] versus 16.0% [8/50]), the overall safety profile revealed significant concerns that warrant careful consideration. The high incidence of pulmonary infections in both groups (48.0% [24/50] versus 40.0% [20/50], respectively) represents substantial morbidity, with nearly half of all patients affected regardless of treatment allocation. Additionally, cerebral herniation occurred in approximately 10% of patients in both groups (12.0% [6/50] versus 10.0% [5/50]), reflecting the severity of the underlying stroke pathology. When evaluating the relationship between adverse events and the intervention, we observed that the lower rate of sICH in the IA-LTH group may be associated with hypothermia’s known effects on blood-brain barrier stabilization and inflammation reduction. The slightly higher incidence of pulmonary infections in the IA-LTH group, though not statistically significant, could potentially be related to mild systemic immunosuppressive effects of cooling or extended procedural time. Cerebral herniation and coagulation abnormalities occurred at similar rates in both groups, suggesting these events were more likely related to the underlying stroke pathology than to the intervention. Notably, no cases of bradycardia were observed in the IA-LTH group (compared to 6.0% [3/50] in controls), supporting our hypothesis that selective brain cooling minimizes systemic hypothermic complications. These findings underscore that while IA-LTH may offer certain safety advantages such as reduced hemorrhagic complications, the overall morbidity profile remains substantial and necessitates careful patient selection and enhanced safety monitoring in future larger-scale trials.

The results align with our hypothesis that IA-LTH may induce rapid and targeted brain cooling without the need for invasive temperature monitoring [21,22]. This non-invasive strategy is particularly relevant in the context of “time is brain,” where swift intervention is paramount. Evidence from preclinical and clinical studies strongly supports the hypothesis of selective brain cooling during IA-LTH [16]. Previous preclinical studies have demonstrated that intra-arterial cold saline infusion reduces the temperature of the ischemic hemisphere, achieving the neuroprotective range of 33–34 °C. This cooling effect is confined to the treated hemisphere and is associated with preserved white matter integrity, reduced inflammation, and improved functional outcomes [23,24]. Our findings suggest that IA-LTH may enhance cerebral perfusion and collateral circulation, thereby potentially reducing reperfusion injury—a concept supported by existing literature on the neuroprotective effects of hypothermia in both clinical studies [17,25] and preclinical animal models [26].

Our study’s findings underscore the neuroprotective capabilities of hypothermia in AIS, leveraging its established mechanisms to attenuate the progression of penumbra to infarct core by reducing energy demands and limiting core expansion [27]. This effect is underpinned by a decrease in cerebral metabolic rate of 7–10% per degree Celsius reduction, a primary mechanism that reflects the brain’s diminished metabolic activity [28–30]. Post-stroke, hypothermia delays ATP depletion and hypoxic depolarization, prevents excitatory neurotransmitter release, curbs inflammation, and maintains the blood-brain barrier’s integrity, thereby mitigating the no-flow phenomenon and enhancing the benefits of acute interventions [31].

Mild hypothermia’s efficacy extends to modulating the inflammatory response by inhibiting leukocyte adhesion and infiltration, as evidenced by reduced expression of endothelial adhesion molecules and leukocyte invasion, which are crucial for preserving the blood-brain barrier and reducing inflammation [31]. Furthermore, by activating the ERK1/2 pathway in MAPK pathways, mild hypothermia promotes neuroprotection through decreased ICAM-1 levels, highlighting its regulatory role in beneficial signaling over deep hypothermia [32,33]. Collectively, these mechanisms exemplify the nuanced yet potent influence of hypothermia in neuroprotection, offering a strategic approach to enhance acute stroke treatment.

The integration of IA-LTH with endovascular thrombectomy (EVT) presents a synergistic treatment approach. This combined strategy leverages the rapid cooling capabilities of IA-LTH to mitigate the ischemic core while EVT addresses the vascular occlusion. Our study explores this dual-modality intervention, which may offer a more comprehensive neuroprotection than either modality alone. While our study was not designed to identify contradictions, the absence of SAEs directly attributable to the IA-LTH intervention was an unexpected yet welcome finding. This suggests that IA-LTH may be a well-tolerated adjunct to EVT. Moreover, IA-LTH presents several advantages over whole-body and intravenous cooling methods. By delivering cold saline directly to the affected cerebral arteries, it achieves rapid and localized brain hypothermia without inducing systemic hypothermia, thereby reducing the risk of complications such as shivering and hemodynamic instability [34–36]. Additionally, this targeted approach avoids the potential for fluid overload associated with intravenous cooling and can be seamlessly integrated with endovascular procedures like mechanical thrombectomy, enhancing overall treatment efficacy [35,36].

Despite these encouraging results, our study has several limitations that warrant consideration. The primary outcome did not reach statistical significance, which may be due to the sample size being insufficient to detect a definitive benefit. Although this pilot study was not statistically powered to detect significant differences in primary clinical outcomes, the sample size was carefully determined to balance feasibility and safety assessments with the ability to identify meaningful differences and estimate effect sizes. As one of the first multicenter randomized controlled trials investigating IA-LTH, this study enhances the generalizability of findings compared to single-center studies. Previous studies by Chen and colleagues and Wan and colleagues utilized sample sizes ranging from 26 to 142 participants [17,18], demonstrating the feasibility and safety of selective intra-arterial hypothermia. These precedents support the adequacy of our pilot study design and sample size in providing meaningful preliminary data to guide future research. The 90-day ordinal shift analysis also showed a non-significant trend favoring the IA-LTH group (*P* = 0.098). We observed a higher prevalence of pulmonary infections in the IA-LTH group, although this did not translate to differences in overall mortality rates. The higher incidence of pulmonary infections in the IA-LTH group may result from hypothermia-induced immune suppression or sedation-related factors impairing airway protection. Future protocols should focus on optimizing selective brain cooling to minimize systemic effects, maintaining intra-procedural warming to reduce hypothermia-related risks, and enhancing respiratory monitoring during sedation to prevent complications. These limitations underscore the need for larger, adequately powered studies to confirm the efficacy and safety profile of IA-LTH in patients with AIS. Promising numerical differences were observed, including a higher proportion of patients achieving functional independence (mRS 0–1 at 90 days) and a favorable trend in the 90-day ordinal shift analysis. These findings suggest the potential benefit of IA-LTH and justify future multicenter trials with larger sample sizes to validate these results and explore its neuroprotective mechanisms. Additionally, while this pilot study adjusted for key confounders such as age and ASPECTS, the small sample size limited the ability to adjust for other potential confounders, including center effects and case-mix variability, which may influence the outcomes. These factors will be addressed in future larger-scale studies.

In summary, our findings highlight the potential of hypothermia as a neuroprotectant, suggesting it warrants further exploration for clinical application. The pleiotropic mechanisms of action and the targeted cooling provided by selective brain hypothermia represent a notable advancement over systemic hypothermia, potentially reducing many of its associated complications. Moving forward, the focus should remain on refining these techniques and carefully evaluating their impact across a broader spectrum of patients who experienced a stroke, with consideration of the limitations observed in this study.

## Supporting information

S1 Appendix Original protocol (v 1.0). (DOCX)

S2 Appendix Original protocol (v 2.0). (DOCX)

S3 Appendix Supplementary Note: Summary of changes. (DOCX)

S4 Appendix Original statistical analysis plan (v 1.0). (DOCX)

S1 CONSORT Checklist CONSORT 2025 Checklist. (DOCX)

S1 SPIRIT Checklist SPIRIT 2025 Checklist. (DOCX)

S1 Graphical Abstract Created using BioRender. (PDF)

## Abbreviations

AIS: acute ischemic stroke
aRR: adjusted relative risk
ASPECTS: Alberta Stroke Program Early CT Score
CI: confidence interval
CTA: computed tomography angiography
END: early neurological deterioration
EVT: endovascular therapy
IA-LTH: intra-arteria local therapeutic hypothermia
IQR: interquartile range
ISOLATION: Intra-arteria local therapeutic hypothermia in patientS with acute anteriOr circulation LArge vessel occlusion Treated with endovascular reperfusION
ITT: intention-to-treat
MRA: magnetic resonance angiography
mRS: Modified Rankin Scale
NIHSS: National Institutes of Health Stroke Scale
RR: relative risk
SAEs: serious adverse events
sICH: symptomatic intracranial hemorrhage
