# Supplementary material for: Intra-arterial selective hypothermia for acute ischemic stroke neuroprotection: A multicenter pilot trial in China
Source: PLoS Med. 2025 Jul 24;22(7):e1004668. doi: 10.1371/journal.pmed.1004668 (PMC12289068; doi:10.1371/journal.pmed.1004668)
Supplement: S1 Appendix — (DOCX) [file pmed.1004668.s001.docx]

**Original protocol (v 1.0)**

**Intra-arteria local therapeutic hypothermia in patients with acute anterior circulation large vessel occlusion treated with endovascular treatment**

an open label multi-center randomized pilot trial

**Protocol**

| **Sponsored by:** | Guangdong Second Provincial General Hospital |
| --- | --- |
| **Principal investigator** | Zhixin Huang |
| **Co-Sponsored by:** | The First Affiliated Hospital of the University of Science and Technology of China |
| **Co- Principal Investigator** | Wen Sun |
| **Version number：** | V1.0, 24 September 2023 |

Confidentiality Statement:

All rights to the information contained in this proposal belong to the Guangdong Second People's Hospital and First Affiliated Hospital of the University of Science and Technology of China. This information is solely intended for review by researchers, collaborating researchers, ethics committees, supervisory and regulatory bodies, and other relevant medical institutions. Without written approval from the sponsor, it is strictly prohibited to disclose any information to third parties unrelated to this study, except for necessary explanations provided to potential participants who may be involved in this research when obtaining their informed consent.

[1. Study Synopsis 4](#_Toc21986)

[Schedule of Assessments 8](#_Toc4505)

[1. Background 9](#_Toc25022)

[2. Trial Objectives 14](#_Toc3240)

[3. Trail Design 14](#_Toc2636)

[4. Patient Population 15](#_Toc25828)

[**4.1. Inclusion criteria 15**](#_Toc11905)

[**4.2. Exclusion criteria 15**](#_Toc2481)

[5. Treatment of subjects 17](#_Toc17278)

[**5.1. Detail Operating Procedures 17**](#_Toc6572)

[**5.2. Selection of Operating Equipment 17**](#_Toc6989)

[6. Sample size calculation 18](#_Toc7450)

[7. Randomization 18](#_Toc2583)

[8. Outcomes 19](#_Toc6308)

[**8.1. Primary Efficacy Outcome 19**](#_Toc9822)

[**8.2. Secondary Efficacy Outcomes 19**](#_Toc25412)

[**8.3. Safety Outcomes 19**](#_Toc967)

[9. Withdrawal of individual subjects 20](#_Toc16462)

[10. Premature termination of the study 20](#_Toc1398)

[11. Safety reporting 20](#_Toc1779)

[**11.1. Temporary halt for reasons of subject safety 20**](#_Toc3086)

[**11.2. AEs and SAEs 20**](#_Toc10934)

[**11.3. Follow-up of adverse events 22**](#_Toc9415)

[**11.4. Data Safety Monitoring Board (DSMB) 22**](#_Toc21670)

[12. Statistical analysis 23](#_Toc19014)

[13. Interim Analysis 24](#_Toc26851)

[14. Ethical considerations 24](#_Toc18815)

[15. Administrative aspects, monitoring and publication 27](#_Toc20986)

[**15.1. Handling and storage of data and documents 27**](#_Toc31335)

[**15.2. Monitoring and quality assurance 27**](#_Toc532)

[**15.3. Amendment 27**](#_Toc17758)

[**15.4. Annual progress report 28**](#_Toc5376)

[**15.5. Temporary halt and (prematurely) end of study report 28**](#_Toc8261)

[**15.6. Public disclosure and publication policy 28**](#_Toc31719)

[16. Reference 29](#_Toc27870)

[Appendix 1：Alberta Stroke Program Early CT Score (ASPECTS) 33](#_Toc22997)

[Appendix-2 Modified Treatment in Cerebral Ischemia (mTICI) scale 33](#_Toc12558)

[Appendix-3 Modified Rankin Scale (MRS) 34](#_Toc30715)

**1. Study Synopsis**

| **Study Title** | | Intra-arteria local therapeutic hypothermia in patients with acute anterior circulation large vessel occlusion treated with endovascular treatment: an open label multi-center randomized pilot trial |
| --- | --- | --- |
| **Sponsor** | | Guangdong Second Provincial General Hospital  The First Affiliated Hospital of the University of Science and Technology of China |
| **Indication** | | Acute anterior circulation large vessel occlusive stroke |
| **Number of Subjects** | | The clinical trial plans to enroll 100 subjects, with 50 subjects in the treatment group and 50 subjects in the control group. |
| **Study Design** | | Prospective, multicenter, randomized controlled, pilot study |
| **Study Duration** | | 4 months |
| **Study Population** | | Patients who experienced a stroke with acute terminal internal carotid artery and/or middle cerebral artery M1 segment occlusion |
| **Trial Objectives** | | This study was designed to evaluate the efficacy and safety of mechanical thrombectomy (MT) combined with selective intra-arterial cooling infusion in patients with acute anterior circulation large artery occlusion. |
| **Randomization** | | Participants will be randomized in a 1:1 ratio using a stratified randomization method based on age and ASPECTS of the central network randomization system to MT combined with selective intra-arterial cooling infusion (hypothermia group) or MT combined with normal temperature saline infusion (normal temperature group) |
| **Inclusion Criteria** | | 1. Age ≥ 18 years and ≤ 80 years. 2. Acute focal or generalized neurological deficits. 3. Acute occlusion of the terminal segment of the internal carotid artery and/or the M1 segment of the middle cerebral artery confirmed by magnetic resonance angiography (MRA) or computed tomography angiography (CTA). 4. There is a significant likelihood that the vascular occlusion is causally related to the neurological deficits. 5. Symptoms onset to the anticipated puncture time within 6 hours, or within 16 hours if meeting the imaging criteria of DAWN and DEFUSE3, or within 24 hours if meeting the imaging criteria of DAWN. 6. Based on clinical judgment, the approach path is reasonable, and the surgical and procedural instruments can be successfully navigated to the lesion. 7. The patient or their guardian understands the purpose of the trial, voluntarily agrees to participate, signs a written informed consent form, and is capable of undergoing clinical follow-up. |
| **Exclusion Criteria** | | 1. Modified Rankin Scale (MRS) score > 1 prior to the onset of the disease. 2. Acute infarcts present simultaneously in both cerebral hemispheres and/or in both the anterior and posterior circulations. 3. National Institutes of Health Stroke Scale (NIHSS) score < 6. 4. Presence of intracerebral hemorrhage or subarachnoid hemorrhage confirmed by CT or MRI. 5. Active bleeding, severe anemia, or coagulation disorders. (One or more of the following laboratory findings: hemoglobin < 10 g/dL, platelet count < 100,000/µL, uncorrected INR > 1.5, PT > 1 minute above normal limits, or heparin-induced thrombocytopenia). 6. Cardiac function greater than Class I, or a history of significant acute or chronic heart failure with a substantial risk of acute heart failure or intolerance to fluid infusion as judged by the clinician. 7. Severe cardiac, hepatic, or renal diseases. 8. Concurrent malignant tumors or other malignancies with a life expectancy of less than 3 months. 9. Participation in other clinical trials, whether in the study phase or follow-up phase. |
| **Treatments** | | Patients were divided into Mechanical thrombectomy (MT) combined with hypothermia group or normal temperature group. |
| **Consent** | | Explicit written, signed informed consent from the subject or legally authorized representative will be obtained prior to any protocol specific procedures. |
| **Effective**  **Endpoints** | **Primary**  **Endpoint** | 90 days (±7 days) modified Ranking Scale (mRS) 0-2  The mRS, a global measure of disability, comprises of seven grades ranging from 0 (no symptoms) to 6 (death). |
|  | **Secondary**  **Endpoints** | 1. mRS score 0-1 at 90-day follow-up 2. Rate of successful reperfusion 3. Infarct Volume on CT or MRI DWI Imaging (Postoperative 24 Hours to 7 Days) 4. Rate of recurrent occlusion on MRA or CTA (postoperative 24 Hours to 7 Days) 5. Early neurological functional changes within 24 hours 6. Perioperative rectal temperature variation |
| **Safety**  **Endpoints** | | 1. Rate of symptomatic intracranial hemorrhage within 72 hours (Heidelberg Bleeding Classification) 2. Rate of coagulation disorders within 7 days 3. Incidence of pulmonary Infection 4. Incidence of new-onset bradycardia during the perioperative period |
| **Sample Size**  **Calculation** | | This study is a pilot study aiming to include 50 subjects in the treatment group and 50 subjects in the control group. Based on the results of the pilot study, the sample size for a subsequent multicenter randomized controlled trial will be determined. |

# Schedule of Assessments

|  | Baseline | 0 d | 24 (±12) hours | 72 (±12) hours | 7±1 day  /at discharge  visit | 90-day  (±7 days)  visit |
| --- | --- | --- | --- | --- | --- | --- |
| Informed Consent Form Present Illness | **×** |  |  |  |  |  |
| Medical history | **×** |  |  |  |  |  |
| Prior medication | **×** |  |  |  |  |  |
| Preoperative Diagnosis | **×** |  |  |  |  |  |
| Vital Signs | **×** | **×** | **×** | **×** | **×** |  |
| NIHSS score | **×** |  | **×** | **×** | **×** |  |
| mRS score | **×** |  |  |  | **×** | **×** |
| ASPECT score | **×** |  |  |  |  |  |
| Laboratory examination |  |  | **×** | **×** |  |  |
| Head + Chest CT | **×** |  |  | **×** |  |  |
| CTA or MRI+MRA | **×** |  |  | **×** |  |  |
| ECG |  |  |  |  |  |  |
| DSA | **×** | **×** |  |  |  |  |
| Inclusion and Exclusion Criteria | **×** |  |  |  |  |  |
| Randomization |  | **×** |  |  |  |  |
| Surgical Procedure Details | **×** | **×** |  |  |  |  |
| Postoperative Medication | **×** | **×** | **×** | **×** | **×** | **×** |
| Adverse event | **×** | **×** | **×** | **×** | **×** | **×** |

# Background

Stroke has become the leading cause of death among residents in China, with acute ischemic stroke (AIS) accounting for approximately 60% to 80% of cases ^[1]^. According to the MONICA study by the World Health Organization, the incidence of stroke in China is increasing annually at an average rate of 8.7% ^[2]^. AIS has emerged as a primary disease that threatens human health and life, imposing significant burdens on families, individuals, and society as a whole.

In the management of AIS, intravenous thrombolysis is the most effective method in the ultra-early treatment. However, thrombolysis has strict time window limitations, and its prognosis for AIS caused by large vessel disease is poor, resulting in less than 3% of patients benefiting from thrombolysis. Among all AIS cases, large vessel occlusion accounts for approximately 28% to 46%, often indicating a poorer prognosis and relatively lower survival rates ^[3]^.

Mechanical thrombectomy (MT) has been proven to be safe and effective, playing a crucial role in the modern management of AIS. In recent randomized clinical trials, the new generation stent retrievers have achieved high success rates (66%-94%) in intracranial large vessel occlusive stroke revascularization ^[4-8]^. However, only 46% of patients treated with MT achieved functional independence at 90 days, with approximately 15% of patients dying within 90 days post-treatment. Therefore, new adjunctive treatment strategies are needed to further improve the clinical outcomes of patients with AIS undergoing MT.

Research on neuroprotection has a long history, with investigators evaluating thousands of neuroprotective strategies, including neuroprotective drugs, hypothermia therapy, among others. Among these, therapeutic hypothermia (TH) is considered to be a relatively effective approach ^[9]^.

TH is an important component of low-temperature therapy, which primarily reduces the patient's whole-body or local temperature by physical means to achieve therapeutic effects. Therapeutic hypothermia is classified clinically into mild hypothermia (33-35°C), moderate hypothermia (28-32°C), deep hypothermia (17-27°C), and profound hypothermia (4-16°C), with mild and moderate hypothermia (28-35°C) collectively referred to as mild-to-moderate hypothermia ^[10]^.

TH can effectively reduce the mortality of sudden cardiac arrest, neonatal hypoxic brain injury, and significantly improve neurological function ^[11-13]^. Favorable therapeutic effects have also been achieved using mild-to-moderate hypothermia in acute traumatic brain injury and cardiac bypass surgery ^[14,15]^. Therefore, mild-to-moderate hypothermia holds great promise in the treatment of ischemic stroke.

However, despite promising results, as many as 39% of treated patients still experience some adverse events related to whole-body hypothermia, including pneumonia and arrhythmias. A multicenter, randomized, phase III clinical trial in Europe aimed to assess whether initiating active whole-body cooling to 34-35°C within 6 hours of acute ischemic stroke onset and maintaining it for 12-24 hours, as opposed to standard treatment, could improve patients' functional outcomes at 90 days. However, the trial, which initially planned to include 1500 patients, only enrolled 98 patients in the end and failed to demonstrate an impact of whole-body TH on patients' 90-day functional outcomes. Moreover, among patients randomly assigned to receive whole-body TH, only one-third achieved the intended cooling target ^[16]^, largely due to increased occurrence of adverse events associated with whole-body TH, including shivering, bradycardia, hypothermia-associated pneumonia, hypertension, congestive heart failure, hyponatremia, hypokalemia, hypomagnesemia, hypoxemia, hypercapnia, and acidosis ^[17-20]^. Therefore, the necessity of assuming the risk of adverse events associated with whole-body hypothermia for the sake of its neuroprotective effects warrants careful consideration. Clarifying the balance of benefits and risks in such patients holds significant clinical relevance.

Arterial infusion of cold saline directly targets ischemic brain tissue, avoiding the need to lower core body temperature and its associated systemic side effects. This method provides a more ideal solution: under imaging guidance, a microcatheter is used to guide 4°C saline directly to the infarct site through the blood vessels, lowering the temperature only in the ischemic area. This achieves the goal of local therapeutic hypothermia (LTH) ^[21, 22]^. Arterial infusion of cold saline is a novel cooling therapy that leverages the high blood flow in the brain to directly infuse low-temperature fluid into the target arteries of the brain, enabling rapid, selective cooling. Thermodynamic models demonstrate that arterial infusion of cold saline can cool 300g of brain tissue per minute, with a cooling rate of 1°C/min ^[23]^, which is 10-20 times faster than systemic venous cooling and 18-42 times faster than surface cooling, which takes 3-7 hours ^[24]^.

The precise targeted cooling method can serve as an adjunctive therapy for MT. During the surgical procedure, a microcatheter used for deploying the stent retriever is coaxially advanced into the femoral artery via a guiding catheter. The microcatheter traverses upward through the neck until it passes the clot causing ischemic symptoms. Using a 10ml/min flow rate, 50ml of 0.9% saline is injected into the ischemic region through the microcatheter, allowing for pre-reperfusion cooling of the area. Following MT, upon confirming vascular patency via digital subtraction angiography (DSA), 4°C saline is infused into the ischemic brain tissue at a rate of 30mL/min, for 10 minutes, achieving LTH. This approach is straightforward and mitigates the systemic side effects associated with whole-body cooling, offering broad prospects for clinical application ^[25]^.

Konstas et al. ^[26]^ confirmed through a computer-simulated model of cerebral infarction that perfusing isotonic saline at 2-3°C into the internal carotid artery at a rate of 30 ml/min could reduce the temperature of the ipsilateral brain to hypothermic levels within 10 minutes, with only a slight decrease in core body temperature and minimal systemic adverse reactions. A follow-up study of 18 patients with intracranial arteriovenous malformations revealed that perfusing isotonic saline at 4-7°C into one side of the internal carotid artery at a rate of 33 ml/min for 10 minutes reduced the jugular venous bulb temperature (considered representative of brain tissue temperature) by 0.84°C, with only a slight decrease in core body temperature (0.15°C) and no significant changes in vital signs and parameters reflecting general physiological conditions such as hematocrit. This suggests that selective hypothermic treatment via selective arterial infusion is feasible and safe for cerebral cooling in humans ^[27]^.

A recent preliminary study by Chen et al. ^[28]^ investigated the feasibility and safety of selective intracranial hypothermia in acute ischemic stroke using intra-arterial infusion of physiological saline combined with mechanical thrombectomy. The study demonstrated the safety and feasibility of perfusing cold physiological saline (4°C) into the revascularized vascular territory via a catheter during the procedural intervention.

In a prospective non-randomized cohort study, 113 patients with acute large artery occlusive stroke were consecutively enrolled. Among them, 45 patients received intra-arterial cold saline infusion combined with MT while 68 patients received MT alone. Results demonstrated no significant differences between the two groups in terms of vital signs, key laboratory values, symptomatic and any intracranial hemorrhage, coagulation abnormalities, pneumonia, urinary tract infections, and mortality. Adjusted regression analysis indicated a significant intergroup difference favoring the hypothermia group in final infarct volume (FIV) assessed by CT within 3-7 days postoperatively. At 90 days, no differences were observed in the proportion of patients achieving functional independence (mRS score 0-2).

The study demonstrates that in patients with AIS, the combination of intra-arterial cold saline infusion with MT for LTH is feasible and safe. However, due to limitations of the cohort study, certain unknown variables and selection bias that may affect the results cannot be excluded; thus, the efficacy of intra-arterial cold saline infusion combined with MT remains undetermined. Furthermore, there is a lack of large-scale randomized controlled trials internationally to confirm the efficacy of intra-arterial cold saline infusion. Therefore, we are conducting this prospective, multicenter, randomized controlled trial using blinded endpoint assessment to evaluate whether the use of intra-arterial cold saline infusion for LTH can improve the neurological functional prognosis in patients who experienced an acute large vessel occlusive stroke after MT, to explore the effectiveness and safety of intra-arterial cold saline infusion, and to promote the development of cerebrovascular intervention techniques, thereby advancing the clinical translation of therapeutic hypothermia.

# Trial Objectives

The objective of this study is to evaluate the efficacy and safety of selective intra-arterial cooling infusion combined with MT in patients with acute anterior circulation large artery occlusion.

To evaluate the efficacy of MT combined with hypothermia group or normal temperature group: (1) 90 days mRS 0-2; (2) Rate of successful reperfusion; (3) Infarct Volume on CT or MRI DWI Imaging (postoperative 24 hours to 7 days); (4) Rate of Recurrent Occlusion on MRA or CTA (Postoperative 24 Hours to 7 Days); (5) Early neurological functional changes within 24 hours; (6) Perioperative rectal temperature variation.

To evaluate the safety of hypothermia group compared with normal temperature group. The measures of safety will be the rates of (1) symptomatic intracranial hemorrhage within 72 hours; (2) Rate of coagulation disorders within 7 days; (3) Incidence of pulmonary Infection; (4) Incidence of new-onset bradycardia during the perioperative period

# Trail Design

Intra-arteria local therapeutic hypothermia in patients with acute anterior circulation large vessel occlusion treated with endovascular treatment: an open label multi-center randomized pilot trial, aiming to evaluate the efficacy and safety of selective intra-arterial cooling infusion combined with MT in patients with acute anterior circulation large artery occlusion.


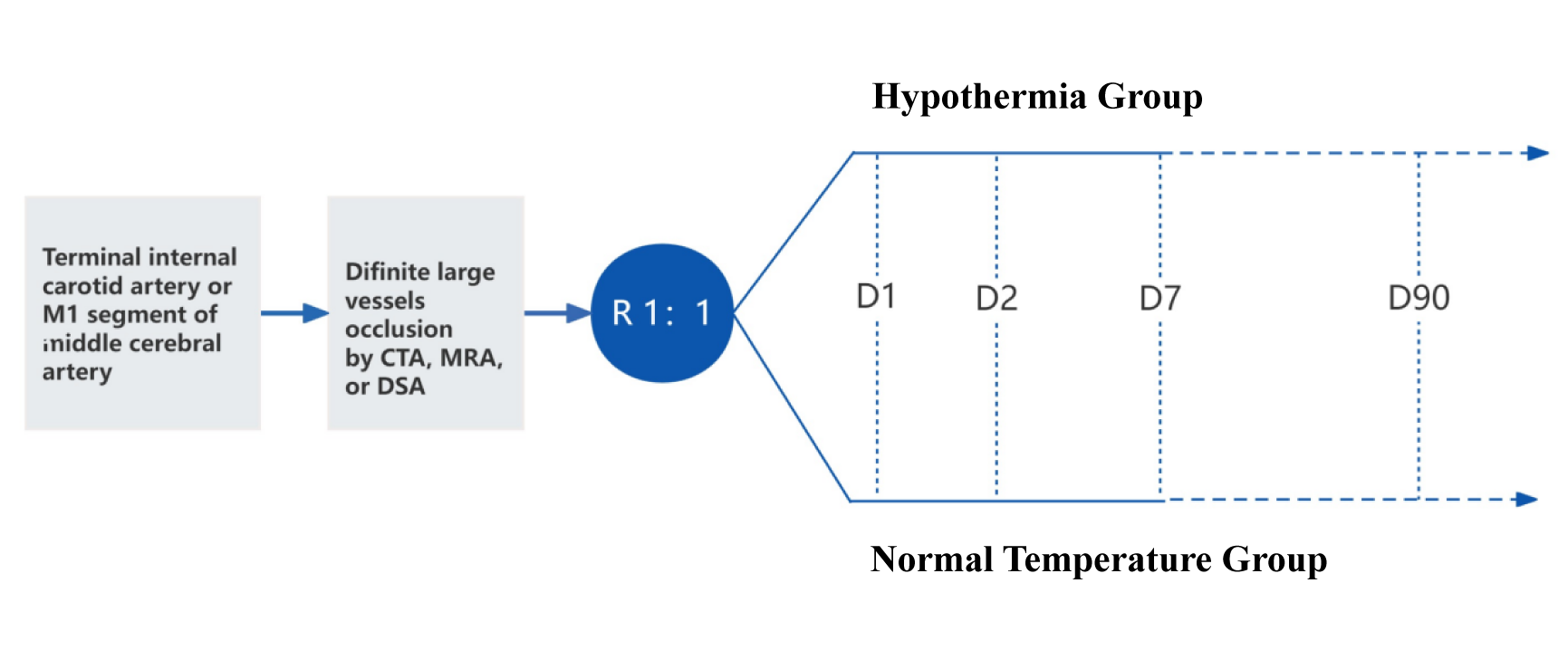


# Patient Population

All participants will undergo a comprehensive neurological and clinical assessment, as well as routine laboratory blood tests and baseline brain imaging. These assessments and tests will be conducted and reviewed by the site physician, including the use of non-contrast CT/CT angiography or diffusion-weighted imaging/MR angiography as part of the standard of care. Each participating site will report the total number of patients admitted with a diagnosis of acute anterior circulation large artery occlusion to monitor enrollment potential. A screening log is not required. Eligible patients identified during routine screening will be provided with necessary information and consent (if required) to participate in the study. Randomized patients will be followed up for the 90-day study period.

## Inclusion criteria

1. Age ≥ 18 years and ≤ 80 years.
2. Acute focal or generalized neurological deficits.
3. Acute occlusion of the terminal segment of the internal carotid artery and/or the M1 segment of the middle cerebral artery confirmed by magnetic resonance angiography (MRA) or computed tomography angiography (CTA).
4. There is a significant likelihood that the vascular occlusion is causally related to the neurological deficits.
5. Symptoms onset to the anticipated puncture time within 6 hours, or within 16 hours if meeting the imaging criteria of DAWN and DEFUSE3, or within 24 hours if meeting the imaging criteria of DAWN.
6. Based on clinical judgment, the approach path is reasonable, and the surgical and procedural instruments can be successfully navigated to the lesion.
7. The patient or their guardian understands the purpose of the trial, voluntarily agrees to participate, signs a written informed consent form, and is capable of undergoing clinical follow-up.

## Exclusion criteria

1. Modified Rankin Scale (MRS) score > 1 prior to the onset of the disease.
2. Acute infarcts present simultaneously in both cerebral hemispheres and/or in both the anterior and posterior circulations.
3. National Institutes of Health Stroke Scale (NIHSS) score < 6.
4. Presence of intracerebral hemorrhage or subarachnoid hemorrhage confirmed by CT or MRI.
5. Active bleeding, severe anemia, or coagulation disorders. (One or more of the following laboratory findings: hemoglobin < 10 g/dL, platelet count < 100,000/µL, uncorrected INR > 1.5, PT > 1 minute above normal limits, or heparin-induced thrombocytopenia).
6. Cardiac function greater than Class I, or a history of significant acute or chronic heart failure with a substantial risk of acute heart failure or intolerance to fluid infusion as judged by the clinician.
7. Severe cardiac, hepatic, or renal diseases.
8. Concurrent malignant tumors or other malignancies with a life expectancy of less than 3 months.
9. Participation in other clinical trials, whether in the study phase or follow-up phase.

# Treatment of subjects

Patients who preliminarily meet the criteria should be screened with informed consent from the patient or legally authorized representative. After meeting the inclusion criteria, randomization should be performed. Patients randomized to the hypothermia group will undergo MT and receive hypothermic saline infusion. Patients randomized to the normal temperature group will undergo MT and receive normal temperature saline infusion.

## Detail Operating Procedures

Randomization of subjects will occur at the start of cerebral vascular intervention treatment. The detailed procedures were left for the operators’ choice. Thrombectomy with combined contact aspiration and stent retriever was recommended for all patients. In the treatment group, after crossing the occluded vessel segment with a microcatheter and confirming its location in the true lumen, 4℃-saline will be extracted from the refrigerator. 50ml will be drawn within 2 minutes and then continuously infused at a rate of 10ml/min using an infusion pump. After infusion, standard thrombectomy procedures will be performed. After completion of the thrombectomy procedure (regardless of achieving 2b/3-grade reperfusion), another 300ml of saline will be extracted within 2 minutes and infused at a rate of 30ml/min for 10 minutes through the intermediate catheter or guiding catheter placed in the internal carotid artery. Post-infusion, contrast-enhanced cerebral angiography using room temperature contrast agent is generally not recommended to dilute the local temperature. (If reperfusion could not be achieved post-procedure, a second step of hypothermic treatment via the guiding catheter or intermediate (aspiration) catheter will be administered). (If the microcatheter cannot pass through the occluded segment or the direct aspiration without the microcatheter was performed for thrombectomy. After completion of the thrombectomy procedure (regardless of achieving 2b/3-grade reperfusion), 350ml of saline will be administered through the guiding catheter or intermediate (aspiration) catheter. The control group will undergo similar procedures but without the use of 4℃ saline, instead using normal temperature saline infusion

To reduce operational differences between centers, prior to the start of the clinical trial, the monitor and responsible persons from each trial center should train the researchers on the trial protocol, clarify the assessment criteria for efficacy and safety indicators, and standardize the researchers' surgical and hypothermic treatment procedures.

## Selection of Operating Equipment

All equipment used in this study, including puncture needles, arterial sheaths, angiographic catheters, guiding catheters, intermediate catheters, guide wires, various types of stents, and thrombectomy devices, must be approved by the National Medical Products Administration. Operators may choose appropriate equipment based on the characteristics of the patient's vascular lesions and should document their choices.

The 4℃-saline used in the hypothermia group must be stored in a thermostatic at 4 ℃ for at least 24 hours.

The normal temperature saline infusion used in the normal temperature group must be stored in a thermostatic at normal temperature for over 24 hours.

# Sample size calculation

This study employed a prospective multicenter randomized open-label parallel controlled pilot trial design. The treatment group received combined arterial selective cold infusion of MT, while the control group received combined room temperature saline infusion of MT. This study is a pilot study aiming to include 50 subjects in the treatment group and 50 subjects in the control group. Based on the results of the pilot study, the sample size for a subsequent multicenter randomized controlled trial will be determined.

# Randomization

This study employs a stratified randomization method for subject randomization. The stratification factors include patient age (divided into age ≥75 and 75>age≥18) and ASPECTS score (stratified by scores ≥8 and <8). After obtaining written informed consent, patients will be randomly allocated in a 1:1 ratio to the hypothermia group or the normal temperature group.

# Outcomes

## Primary Efficacy Outcome

The primary endpoint of this study is 90 days (±7 days) mRS 0-2 after randomization. The primary endpoint of mRS 0-2 at 90 days (±7 days) will be assessed by a local evaluator blinded to the treatment and a central independent Rankin Scale-certified evaluator. In case of disagreement between the obtained mRS scores at each evaluation, the study will consider as correct data the one adjudicated by centra blinded evaluator.

## Secondary Efficacy Outcomes

1. mRS score 0-1 at 90-day follow-up;
2. Rate of successful reperfusion;
3. Infarct Volume on CT or MRI DWI Imaging (postoperative 24 Hours to 7 Days);
4. Rate of recurrent occlusion on MRA or CTA (postoperative 24 Hours to 7 Days);
5. Early neurological functional changes within 24 hours;
6. Perioperative rectal temperature variation

The technical efficacy outcomes regarding recanalization were substantial reperfusion, as assessed by means of catheter angiography in the EVT group and defined as a modified Treatment in Cerebral Infarction score of 2b (50 to 99% reperfusion) or 3 (complete reperfusion).

## Safety Outcomes

# Symptomatic intracranial hemorrhage within 72 hours;

# Rate of coagulation disorders within 7 days;

# Incidence of pulmonary Infection;

# Incidence of new-onset bradycardia during the perioperative period

# Withdrawal of individual subjects

Participants have the right to voluntarily withdraw from the study at any time without any negative consequences. In cases of urgent medical issues, the investigator may decide to exclude a participant from the study. It is essential to emphasize that data from participants who do not provide consent will not be used in the analysis, including the assessment of baseline characteristics.

# Premature termination

The study will only be terminated prematurely if recommended by the Data and Safety Monitoring Board (DSMB). If such a recommendation occurs, the study database will be securely locked after a comprehensive evaluation of the last enrolled patient for a period of 90 days. Following this, the results and findings will be reported diligently.

# Safety reporting

## Temporary halt for reasons of subject safety

If there is substantial evidence suggesting that the ongoing study poses a risk to the health or safety of participants, the study sponsor will take the necessary steps to suspend it. The sponsor will promptly inform all parties involved of the temporary halt and provide a clear explanation for this decision. The study will remain suspended until it undergoes a thorough review by the EC (Ethics Committee). It is the responsibility of the investigators to ensure that all participants are kept well-informed throughout this process.

## AEs and SAEs

### Adverse events (AEs)

Adverse events encompass any undesirable experiences encountered by a subject during the study, regardless of their relationship to the trial procedures. All adverse events, whether spontaneously reported by the subject, observed by the investigator, or their staff, will be diligently documented. Pre-existing illnesses prior to the occurrence of a stroke will not be classified as adverse events unless there is a notable increase in severity or frequency during the investigation. It is important to note that deaths should not be recorded as adverse events, but rather indicated as the outcome of a specific adverse event. Any adverse events experienced by study participants after enrollment (starting from the point of randomization) must be accurately recorded in the Case Report Form.

### Serious adverse events (SAEs)

A serious adverse event (SAE) is defined as any unfavorable medical occurrence or effect that meets the following criteria:

(1) Death

(2) Poses an immediate life-threatening situation at the time of the event

(3) Necessitates hospitalization or prolongs existing hospitalization

(4) Leads to enduring or significant disability or incapacity

(5) Demands medical or surgical intervention to prevent any of the mentioned outcomes

Additionally, any other medically important events that, although did not result in the above outcomes due to medical or surgical intervention, could have done so based on appropriate medical judgment. It is important to note that elective hospital admissions will not be considered as serious adverse events.

Upon detection of a serious adverse event, the investigator will report it immediately to the trial coordinator, who remains accessible 24/7. The following serious adverse events occurring during the study period will be promptly reported to the sponsor upon the investigator's knowledge: any cause death, symptomatic intracranial hemorrhage, extracranial hemorrhage, aspiration pneumonia, allergic contrast reactions, and new ischemic stroke in a different vascular territory.

Technical complications or vascular damage at the target lesion, such as perforation or dissection, that do not result in clinically detectable serious adverse events, as well as neurological deterioration not caused by intracranial hemorrhage or new ischemic stroke but are consistent with the natural course of ischemic stroke and its treatment, will not be immediately reported.

## Follow-up of adverse events

All AEs will be closely monitored and followed up until they have resolved or reached a stable state. Depending on the nature of the event, further tests, medical procedures, or referrals to a general physician or specialist may be necessary. SAEs must be reported throughout the duration of the study, which extends up to 90 days after randomization, as defined as the end of the study period.

## Data Safety Monitoring Board (DSMB)

In order to ensure the safety of study participants and uphold the ethical and scientific integrity of the trial, an independent Data Safety Monitoring Board (DSMB) will be established. The DSMB will evaluate the study data from a third-party and unbiased perspective. It will comprise of clinicians experienced in stroke treatment, a biostatistician, and a neuro-interventionalist, who will monitor the progress of the trial.

The DSMB will convene regularly, at least once a year or after the inclusion of the next 100 patients, whichever occurs first. Its primary responsibility will be to assess the occurrence of adverse events on a center-by-center and procedure-by-procedure basis. The DSMB will analyze the data with the assistance of an independent statistician and make appropriate recommendations and provide advice to the EC based on their findings.

It is important to note that the members of the DSMB will not be involved in authoring the academic paper associated with the study, ensuring an unbiased and objective evaluation process.

# Statistical analysis

1. Data were presented as medians (IQRs) or numbers with percentages. Univariate analysis was performed using the χ2 test for dichotomous variables and independent Student’s t-test or Mann-Whitney U test for continuous variables, as appropriate.
2. For intergroup comparisons of baseline indicators, continuous data will be analyzed using t-tests or Wilcoxon rank-sum tests, while comparisons of categorical data will be conducted using chi-square tests, Fisher's exact test, or Wilcoxon rank-sum tests.
3. In the efficacy analysis, the primary endpoint will be evaluated using logistic regression to assess efficacy based on the intention-to-treat (ITT) principle, calculating the common odds ratio (common OR) for the full analysis set (FAS) data. Additionally, the per-protocol set (PPS) data will be used for sensitivity analysis with the same statistical methods. All statistical analyses will be two-tailed, and a significance level of P<0.05 will be considered statistically significant. For secondary endpoints, center effects will not be considered, and differences in secondary efficacy indicators between the two groups will be compared using chi-square tests or Fisher's exact test.
4. Safety analysis will be based on the Statistical Analysis System (SAS) dataset, describing the incidence rates of safety events in both groups. Logistic regression will be used to compare differences in safety endpoints such as intracranial hemorrhage between the two groups. Various adverse reactions and serious adverse event (SAE) rates between the two groups will be compared using methods such as chi-square tests and Fisher's exact test.

## Subgroup analysis

Subgroup analyses for the primary outcome will be carried out according to:

# Age ≤75 years versus >75 years

# Baseline ASPECTS: <8 versus ≥8

# Ethical considerations

This study adhered to the ethical principles outlined in the Helsinki Declaration. The research protocol and consent forms were submitted to the REB/IRB of each participating hospital. Prior to the commencement of the study, the sponsor was provided with copies of the approval letters from the REB/IRBs, and a record of the membership list of the REB/IRB was maintained.

To ensure that the subjects fully understood the nature of the trial, the investigators were responsible for providing detailed information to the patients or their legal representatives. This included explaining the purpose of the trial, potential benefits and risks, as well as the rights and obligations of the subjects. Patients had the right to withdraw from the study at any time if they chose to do so. Privacy protection for the subjects was ensured throughout the study.

Written informed consent was obtained from each patient or their legal representative prior to their participation in the study. In addition, each patient was required to provide contact information to the investigator at the participating center. Likewise, the investigator provided their own contact number to the patient, ensuring easy communication between them at any time.

The study received ethical approval from the Ethics Committee of each participating center. SAEs were reported to the REB/IRB in accordance with their specific requirements.

## Risk/benefit analysis

### Risks

Possible complications may occur during an interventional procedure. These complications

include but are not limited to the following:

1. Adverse reaction to antiplatelet/ anticoagulation agents or contrast media
2. Air embolism
3. Arteriovenous Fistula
4. Burning sensation
5. Change in mental status
6. Device(s) deformation, collapse, fracture or malfunction
7. Distal thrombus embolization, including to a previously uninvolved territory
8. General discomfort or pain
9. Hematoma and hemorrhage at puncture site
10. Infection
11. Intracranial hemorrhage
12. Ischemia
13. Nausea and/or vomiting
14. Neurologic deterioration including stroke and death
15. Perforation or dissection of the vessels
16. Post procedure bleeding 24
17. Pseudo aneurysm formation
18. Thrombosis (acute and subacute)
19. Vascular occlusion
20. Vascular spasm

### Benefits

While participation in this study does not guarantee any specific benefits, it is possible that undergoing mechanical thrombectomy treatment may enhance blood flow in the affected artery. As a result, patients may experience a reduction in the severity and frequency of ischemic stroke symptoms, leading to less permanent disability.

Moreover, the knowledge and insights gained from conducting this study could potentially be advantageous for individuals facing similar medical conditions in the future. The information gathered may contribute to advancements in the understanding and treatment of such conditions, benefiting a wider population beyond the study participants.

## Compensation for injury

Each participating center has purchased liability insurance. This insurance provides cover for damage to research subjects through injury or death caused by the study.

# Administrative aspects, monitoring and publication

## Handling and storage of data and documents

The collection of trial data will be facilitated through the utilization of a web-based database, which will be accessed and managed by local research personnel. To maintain confidentiality, each subject's records will be assigned a unique study number, ensuring anonymity. The local investigators will maintain a list that links the codes to the corresponding subject names, ensuring proper identification for future reference.

To further safeguard the privacy of the subjects, any documents containing identifying information will be stored separately from the main study database. These documents will be stored as digital files, categorized according to the study number, and securely stored on a protected drive system. Access to these files will be restricted solely to the study coordinator, ensuring that only authorized individuals can access the information.

## Monitoring and quality assurance

Visits by monitors will be scheduled based on the enrollment rate and previous deviations found at each center. Ideally, an inspection visit will be arranged within 5 working days of a center's enrollment. During these visits, the monitor will validate the informed consent process and review the source data for all subjects. The monitoring process will encompass various data sources, including but not limited to in-patient medical records, outpatient medical records, follow-up medical records, imaging materials, and evaluation forms. Additionally, the monitor will assess the accuracy, completeness, and consistency of data entry. To ensure data accuracy, monitors will compare the data entered in the Case Report Forms (CRFs) with the information obtained from the source documents, such as medical records, imaging materials, and evaluation forms, unless otherwise specified in advance.

## Amendment

In the event that any changes to the study protocol are necessary during the study period, an amendment to the protocol should be made only after receiving a favorable opinion from the steering committee. Once the amendment is approved, it is crucial to promptly report the modifications to the investigators at the participating centers. The report should provide comprehensive details and a justification for the proposed changes.

## Annual progress report

Data monitoring in each center is conducted by the clinical research organization. A summary of the data will be prepared by the independent statistician and submit to the DSMB. Information should be provided includes the date of inclusion of the first subject, numbers of subjects included and numbers of subjects that have completed the trial, serious adverse events/ serious adverse reactions, other problems and amendments. The DSMB will submit a summary of the progress of the trial to the steering committee annually.

## Temporary halt and (prematurely) end of study report

The investigator or sponsor is responsible for notifying the executive committee of the study's conclusion within a timeframe of 8 weeks. The end of the study is determined as the last visit of the final patient. In the event of a temporary suspension of the study, the sponsor will immediately inform the executive committee, providing a clear explanation for this action.

If the study is terminated prematurely, the sponsor must notify the executive committee within 15 days, providing the reasons for the premature termination.

Within one year following the conclusion of the study, the investigators or sponsor will submit a final study report to the executive committee and the Competent Authority. This report will include the study's results, as well as any publications or abstracts related to the study.

## Public disclosure and publication policy

The trial will be registered on the official website [www.chictr.org.cn](http://www.chictr.org.cn/). A comprehensive manuscript, outlining the trial and addressing the research objectives, will be prepared and submitted to a clinical journal. Prior to submission, the manuscript will be shared with the sponsors, allowing them one month to review it. However, the sponsors will not have any influence over the content of the manuscript.

Principal investigators can be contacted to request anonymous data from the study. To obtain the data, a detailed description must be provided, clearly stating the aims and methods for which the data will be used. The data will be made available for this purpose at least 18 months after the publication of the main report.

In addition, the data may be shared with non-commercial entities for scientific purposes, including individual patient meta-analyses. Furthermore, the data can be shared with commercial entities for regulatory purposes.

# Reference

1.Cerebrovascular Disease Study Group, Chinese Society of Neurology, Chinese Medical Association. Guidelines for diagnosis and treatment of acute ischemic stroke in China 2014.ZhonghuaShen Jing KeZaZhi, 2015, 48:246-257

2.Benjamin EJ, Blaha MJ, Chiuve SE, et al. Heart Disease and Stroke Statistics-2017 Update: A Report From the American Heart Association [published correction appears in Circulation. 2017 Mar 7;135(10 ):e646

3. [Smith WS, Lev MH, English JD, Camargo EC, Chou M,Johnston SC,Gonzalez G,Schaefer PW,Dillon WP,Koroshetz WJ,Furie KL. Significance of large vessel intracranial occlusion causing acute ischemic stroke and TIA.Stroke,2009, 40:3834-3840.](https://wvpn.ustc.edu.cn/https/77726476706e69737468656265737421fbf952d2243e635930068cb8/kcms/detail/detail.aspx?dbcode=SJWK&filename=SJWK12112900280183&v=MzExNjhvQk1UNlQ0UFFIL2lyUmRHZXJxUVRNbndaZVp0RlNubFVycklLVndSYUJBPU5pZmNaYks2SDlET3BvOUZadU1QRFhRNg==&uid=WEEvREcwSlJHSldSdmVqMDh6c3VGbkMrMXFISitQNGt5VkRMbHpyNndTbz0=$9A4hF_YAuvQ5obgVAqNKPCYcEjKensW4IQMovwHtwkF4VYPoHbKxJw!!)

4.Berkhemer OA, Fransen PS, Beumer D, et al. A randomized trial of intraarterial treatment for acute ischemic stroke. N Engl J Med 2015; 372: 11–20.

5. Campbell BC, Mitchell PJ, Kleinig TJ, et al. Endovascular therapy for ischemic stroke with perfusion-imaging selection. N Engl J Med 2015; 372: 1009–1018.

6. Goyal M, Demchuk AM, Menon BK, et al. Randomized assessment of rapid endovascular treatment of ischemic stroke. N Engl J Med 2015; 372: 1019–1030.

7. Jovin TG, Chamorro A, Cobo E, et al. Thrombectomy within 8 hours after symptom onset in ischemic stroke. N Engl J Med 2015; 372: 2296–2306.

8. Saver JL, Goyal M, Bonafe A, et al. Stent-retriever thrombectomy after intravenous t-PA vs. t-PA alone in stroke. N Engl J Med 2015; 372: 2285–2295.

9. Neuhaus AA, Couch Y , Hadley G, Buchan AM (2017).Neuroprotection in stroke: the importance of collaboration and reproducibility. Brain, 140:2079-2092.

10.Powers, William J et al. “Guidelines for the Early Management of Patients With Acute Ischemic Stroke: 2019 Update to the 2018 Guidelines for the Early Management of Acute Ischemic Stroke: A Guideline for Healthcare Professionals From the American Heart Association/American Stroke Association.” Stroke vol.

11.Nielsen N, Wetterslev J, Cronberg T, Erlinge D, Gasche Y , Hassager C, et al. (2013). Targeted temperature management at 33 degrees C versus 36 degrees C after cardiac arrest. N Engl J Med, 369:2197-2206.

12. Peberdy MA, Callaway CW, Neumar RW, Geocadin RG, Zimmerman JL, Donnino M, et al. (2010). Part 9: post-cardiac arrest care: 2010 American Heart Association Guidelines for Cardiopulmonary Resuscitation and Emergency Cardiovascular Care. Circulation, 122:S768-786.

13. Jacobs SE, Morley CJ, Inder TE, Stewart MJ, Smith KR, McNamara PJ, et al. (2011). Whole-body hypothermia for term and near-term newborns with hypoxic-ischemic encephalopathy: a randomized controlled trial. Arch Pediatr Adolesc Med, 165:692-700.

14. Li P, Yang C. Moderate hypothermia treatment in adult patients with severe traumatic brain injury: a meta-analysis. Brain Inj, 2014, 28(8): 1036-1041.

15. Kim F, Nichol G, Maynard C, et al. Effect of prehospital induction of mild hypothermia on survival and neurological status among adults with cardiac arrest: a randomized clinical trial. JAMA, 2014, 311(1): 45-52.

16. van der Worp, H Bart et al.(2019) Therapeutic hypothermia for acute ischaemic stroke. Results of a European multicentre, randomised, phase III clinical trial. European stroke journal，4(3):254-262.

17.Hemmen TM, Raman R, Guluma KZ, Meyer BC, Gomes JA, Cruz-Flores S, et al. (2010). Intravenous thrombolysis plus hypothermia for acute treatment of ischemic stroke (ICTuS-L): final results. Stroke, 41:2265-2270.

18. Polderman KH, Herold I (2009). Therapeutic hypothermia and controlled normothermia in the intensive care unit: practical considerations, side effects, and cooling methods. Crit Care Med, 37:1101-1120.

19. Wu L, Wu D, Y ang T, Xu J, Chen J, Wang L, et al. (2020). Hypothermic neuroprotection against acute ischemic stroke: The 2019 update. J Cereb Blood Flow Metab, 40:461-481.

20. Zhang J, Liu K, Elmadhoun O, Ji X, Duan Y , Shi J, et al. (2018). Synergistically Induced Hypothermia and Enhanced Neuroprotection by Pharmacological and Physical Approaches in Stroke. Aging Dis, 9:578-589.

21.Ding Y , Li J, Rafols JA, Phillis JW, Diaz FG (2002). Prereperfusion saline infusion into ischemic territory reduces inflammatory injury after transient middle cerebral artery occlusion in rats. Stroke, 33:2492-2498.

22. Ding Y , Y ao B, Zhou Y , Park H, McAllister JP , 2nd, Diaz FG (2002). Prereperfusion flushing of ischemic territory: a therapeutic study in which histological and behavioral assessments were used to measure ischemia-reperfusion injury in rats with stroke. J Neurosurg, 96:310-319.

23. Slotboom J, Kiefer C, Brekenfeld C, Ozdoba C, Remonda L, Nedeltchev K, et al. (2004). Locally induced hypothermia for treatment of acute ischaemic stroke: a physical feasibility study. Neuroradiology,46:923-934.

24. Konstas AA, Neimark MA, Laine AF, Pile-Spellman J (2007). A theoretical model of selective cooling using intracarotid cold saline infusion in the human brain. J Appl Physiol (1985), 102:1329-1340.

25. Wu C, Zhao W, An H, Wu L, Chen J, Hussain M, et al. (2018). Safety, feasibility, and potential efficacy of intraarterial selective cooling infusion for stroke patients treated with mechanical thrombectomy. J Cereb Blood Flow Metab, 38:2251-2260.

26. KonstasAA, NeimarkMA, LaineAF, et al. A theoretical model of selective cooling using intracarotid cold saline infusion in the human brain[J]. J Appl Physiol (1985), 2007, 102(4):1329-1340.

27. ChoiJH, MarshallRS, NeimarkMA, et al. Selective brain cooling with endovascular intracarotid infusion of cold saline: a pilot feasibility study[J]. AJNR Am J Neuroradiol, 2010, 31(5):928-934.

28. ChenJ, LiuL, ZhangH, et al. Endovascular hypothermia in acute ischemic stroke: Pilot study of selective intra-arterial cold saline infusion[J]. Stroke, 2016, 47(7):1933-1935.

# Appendix 1：Alberta Stroke Program Early CT Score (ASPECTS)

The Alberta Stroke Program Early CT Score (ASPECTS) is a semiquantitative method of estimation of infarct size with non-contrast CT during the acute phase. The territory of the middle cerebral artery is allotted 10 points. 1 point is subtracted for an area of early ischaemic change, such as focal swelling, or parenchymal hypoattenuation, for each of the defined regions. A normal CT scan has an ASPECTS value of 10 points. A score of 0 indicates diffuse ischaemia throughout the territory of the middle cerebral artery.
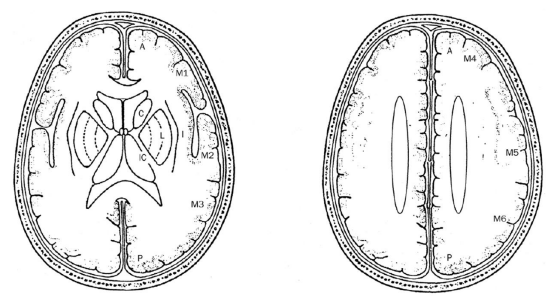


# Appendix-2 Modified Treatment in Cerebral Ischemia (mTICI) scale

| Grades Definitions | |
| --- | --- |
| 0 | no perfusion |
| 1 | antegrade reperfusion past the initial occlusion,but limited distal branch filling |
|  | with little or slow distal reperfusion |
| 2a | antegrade reperfusion of less than half of the occluded target artery previously |
|  | ischemic territory |
| 2b | antegrade reperfusion of more than half of the previously occluded target artery |
|  | ischemic territory |
| 3 | complete antegrade reperfusion of the previously occluded target artery ischemic |
|  | territory,with absence of visualized occlusion in all distal branches |

# Appendix-3 Modified Rankin Scale (MRS)

| 0 No symptoms at all |
| --- |
| 1 No significant disability despite symptoms:able to carry out all usual duties and activities |
| 2 Slight disability:unable to carry out all previous activities but able to look after own affairs without assistance |
| 3 Moderate disability:requiring some help,but able to walk without assistance |
| 4 Moderately severe disability:unable to walk without assistance,and unable to attend to own bodily needs without assistance |
| 5 Severe disability:bedridden,incontinent,and requiring constant nursing care and attention |
| 6 Death |

# Appendix-4 outcome definition

1. Successful reperfusion: Successful reperfusion was defined as a modified thrombolysis in cerebral infarction score >= 2b
2. Recurrent occlusion: recurrent occlusion after successful MT was assessed at 24-hours-7days, and defined as the original occluded site or distal blood vessel experiences reocclusion.
3. Symptomatic intracranial hemorrhage: Symptomatic ICH (sICH) was diagnosed if the newly observed ICH on imaging was related to any of the following conditions: (1) an NIHSS score that increased >4 points; (2) an NIHSS score that increased >2 points in a category; (3) the deterioration led to hemicraniectomy, external ventricular drain placement, intubation, or other major medical interventions. There were no other explanations for the symptom deteriorations.
4. Intracranial Hemorrhage (ICH) was evaluated according to the Heidelberg Bleeding Classification
5. Coagulation disorders: Coagulation disorders refer to medical conditions characterized by abnormalities in the blood clotting process, which can result in either excessive bleeding or abnormal clot formation.
6. Pulmonary Infection: Any instance of lung infection occurring post-procedure, which is a critical safety parameter, especially in the population under study.
7. New-onset bradycardia: new-onset bradycardia is a slower than normal heart rate (below 60 beats per minute (bpm)) that develops suddenly or for the first time.
8. Early Neurological Deterioration: We define early neurological deterioration as an increase of more than 4 points on the National Institutes of Health Stroke Scale (NIHSS) within the first 24 hours following the intervention. This definition specifically excludes cases where the deterioration is attributable to cerebral hemorrhage, ensuring that the assessment accurately reflects the impact of the intervention on neurological status.
