# Supplementary material for: Intra-arterial selective hypothermia for acute ischemic stroke neuroprotection: A multicenter pilot trial in China
Source: PLoS Med. 2025 Jul 24;22(7):e1004668. doi: 10.1371/journal.pmed.1004668 (PMC12289068; doi:10.1371/journal.pmed.1004668)
Supplement: S3 Appendix — (DOCX) [file pmed.1004668.s003.docx]

**Supplementary Note: Summary of changes**

| Sections | Protocol version 1.0 change from | Protocol version 2.0 change to | Justification for Modification |
| --- | --- | --- | --- |
| Methods (Inclusion Criteria) | Age ≥ 18 years and ≤ 80 years. | Age ≥ 18 years and ≤ 85 years. | Inclusion criteria were expanded. |
| Methods (exclusion criteria ) | National Institutes of Health Stroke Scale (NIHSS) score ≤ 6. | Cancel National Institutes of Health Stroke Scale (NIHSS) score ≤ 6. | Inclusion criteria were expanded. |

The following is a list of main protocol changes from protocol version 1.0 dated 24 September 2023 to version 2.0 dated 10 October 2023. The main reasons for the protocol changes in this series of amendments are:

**Note:** 1. Age Criterion: The original protocol criterion "≥18 years and ≤80 years" was modified to "≥18 years and ≤85 years." Considering the aging population and high stroke incidence in the elderly, this modification enhanced the external validity and clinical relevance of our study. 2. We removed the exclusion criterion "NIHSS score ≤6" from the original protocol, which allowed us to include patients with lower NIHSS scores. This modification was based on evidence that patients with low NIHSS scores but large vessel occlusions may still benefit from endovascular therapy.
