# Supplementary material for: Intra-arterial selective hypothermia for acute ischemic stroke neuroprotection: A multicenter pilot trial in China
Source: PLoS Med. 2025 Jul 24;22(7):e1004668. doi: 10.1371/journal.pmed.1004668 (PMC12289068; doi:10.1371/journal.pmed.1004668)
Supplement: S4 Appendix — (DOCX) [file pmed.1004668.s004.docx]

**Original statistical analysis plan (v 1.0)**

**Intra-arteria local therapeutic hypothermia in patients with acute anterior circulation large vessel occlusion treated with endovascular treatment: an open label multi-center randomized pilot trial**

**(ISOLATION)**

**STATISTICAL ANALYSIS PLAN**

[www.chictr.org.cn](http://www.chictr.org.cn) **registration number: ChiCTR2300074990**

Protocol version and date: Version 1.0, 24^th^ September 2023

**ABBREVIATIONS**

| **Abbreviation** | **Explanation** |
| --- | --- |
| AE | Adverse Event |
| AIS | Acute Ischaemic Stroke |
| CI | Confidence Interval |
| CRF | Case Report Form |
| IA-LTH | Intra-arteria Local Therapeutic Hypothermia |
| IDMC | Independent Data Monitoring Committee |
| ITT | Intent-to-Treat |
| LAO | Large Artery Occlusion |
| mRS | Modified Rankin Scale |
| NIHSS | National Institute of Health Stroke Scale |
| RR | Relative Rate |
| PP | Per-protocol |
| SAE | Serious Adverse Event |
| SAP | Statistical Analysis Plan |
| TMG | Trial Management Group |

**INTRODUCTION**

Purpose of the statistical analysis plan

The purpose of this Statistical Analysis Plan (SAP) is to delineate the outcome measures, selection of statistical methodologies, and analytical approaches that will be employed to address the study's primary and secondary objectives. This is within the framework of a prospective, multicenter, randomized, open-label, blinded endpoint trial designed to investigate the efficacy and safety of intra-arteria local therapeutic hypothermia (IA-LTH) for patients presenting with acute moderate ischemic stroke due to large artery occlusion (LAO) within a 24-hour window from symptom onset.

Background to the study

Acute ischemic stroke (AIS) is a major global health threat, causing substantial disability and death^1^. Despite the availability of reperfusion therapies, many patients do not regain functional independence^2^, highlighting a critical need for neuroprotective strategies. The "no-reflow" phenomenon after reperfusion, which can worsen ischemic injury, further complicates treatment^3^.

Therapeutic hypothermia has shown promise in other medical emergencies but has not been widely integrated into AIS treatment due to its systemic side effects^4, 5^. An innovative approach, IA-LTH, aims to induce targeted brain cooling to minimize these complications. Preliminary studies hint at potential benefits in reducing infarct volume, yet the impact on patient outcomes is not fully understood.

The ISOLATION trial addresses this gap, evaluating the safety and efficacy of IA-LTH in patients with AIS undergoing mechanical thrombectomy. This study explores whether IA-LTH, when combined with reperfusion therapy, can improve neurological outcomes by reducing reperfusion injury, offering a new frontier in neuroprotection for AIS.

**STUDY OBJECTIVES AND OUTCOMES**

Study Objectives

**Primary Objective**

To test the hypothesis that IA-LTH, when administered as an adjunct to reperfusion therapy, exerts a significant neuroprotective effect on patients with acute ischemic stroke, leading to improved functional outcomes at 90 days, and demonstrates a favorable safety and tolerability profile.

**Secondary Objectives**

1. To determine the proportion of patients achieving excellent functional outcomes at 90 days after treatment with IA-LTH compared to standard care.

2. To evaluate the distribution of modified Rankin Scale (mRS) scores at 90 days by treatment group, assessing the efficacy of IA-LTH.

3. To assess the occurrence of early neurological deterioration within 24 hours post-treatment by treatment group, examining the impact of IA-LTH.

4. To establish all-cause mortality at 90 days by treatment group, evaluating the safety profile of IA-LTH in Patients with AIS.

5. To determine IA-LTH-related safety outcomes by treatment group, specifically examining the tolerability and adverse events associated with IA-LTH.

6. To determine the rate of successful reperfusion in patients treated with IA-LTH compared to those receiving standard care.

7. To assess the infarct volume.

8. To determine the rate of recurrent occlusion detected on MRA or CTA within the postoperative period of 24 hours to 7 days following treatment.

9. To evaluate perioperative rectal temperature variation.

10. To assess the rate of coagulation disorders within the first 7 postoperative days.

11. To evaluate the rate of symptomatic intracranial hemorrhage within 72 hours using the Heidelberg Bleeding Classification.

**Outcomes Primary**

**Outcome**

The primary outcome of our study is the occurrence of a modified Rankin Scale (mRS) score of 0-2 at 90 days post-randomization, which is considered a favorable functional outcome. This binary outcome is defined as a score within the range of 0 to 2 on the mRS, evaluating the degree of neurological disability. The assessment is performed in person by trained evaluators or, if an in-person visit is not feasible, by personnel certified in the scoring of the mRS through telephone interviews at 90 days after randomization. This approach ensures a standardized and reliable measurement of the patients' functional status following the intervention with IA-LTH.

**Secondary outcomes**

1. Excellent functional outcomes at 90 days post-treatment with IA-LTH, compared to standard care, are defined by a mRS score of 0 to 1.

2. Successful reperfusion will be defined as a modified Thrombolysis in Cerebral Infarction (mTICI) score of 2b or 3, as determined by an independent core laboratory blinded to treatment allocation.

3. Infarct volume will be measured on CT or MRI DWI imaging obtained between 24 hours and 7 days postoperatively, as assessed by an independent core laboratory blinded to treatment allocation.

4. Recurrent occlusion will be defined as any new or worsening vessel blockage detected on MRA or CTA between 24 hours and 7 days postoperatively, as assessed by an independent core laboratory blinded to treatment allocation.

5. Occurrence of early neurological deterioration (END) (binary outcome): Early neurological deterioration is defined by an increase of 4 or more points on the NIHSS within 24 hours that is not attributable to intracerebral hemorrhage.

6. Perioperative rectal temperature variation will be defined as the change in rectal temperature before and after treatment.

**Safety Outcomes**

Occurrence of safety outcomes (binary outcome): Safety is monitored through the incidence of adverse events and serious adverse events during the intervention, including but not limited to symptomatic intracranial hemorrhage, coagulation disorders, pulmonary infections, and new-onset bradycardia.

**﻿**

**STUDY DESIGN**

**Design**

This is a prospective, random, open label, blinded end point, multi-center trial in patients with acute ischemic stroke.

**Randomization**

Participants will be randomized in a 1:1 ratio using a stratified randomization method based on age and ASPECTS of the central network randomization system to MT combined with IA-LTH (hypothermia group) or MT combined with normal temperature saline infusion (normal temperature group)

**Sample Size**

For this pilot study, a formal sample size calculation was not conducted, as there was a lack of pertinent data from prior randomized controlled trials (RCTs). Consequently, the sample size, consisting of 50 patients per group, was primarily based on the recommendations provided by the Steering Committee.

**ANALYSIS POPULATIONS**

**Study population data sets**

The composition of each analysis set will be ascertained, recorded, and the exclusion criteria will be detailed before the database is finalized. A descriptive summary table will categorize participants by their treatment group and outline any instances of protocol deviation or non-compliance.

**Intent-to-Treat (ITT) population**

All individuals providing valid consent will be considered part of the ITT population, aligned with their randomized treatment assignment, even if they discontinue treatment early or deviate from the protocol. Those who are lost to follow-up or who withdraw are excluded from the ITT analysis.

If crossover occurs between groups, the following analysis methods will also be required:

**As-Treated (AT) Population**

A group that includes participants based on the treatment they actually received, regardless of their initial randomization or protocol adherence. This population is used to analyze the effects of treatment as administered, rather than as planned, and may include participants who did not complete the full course of treatment.

**Per-Protocol (PP) Population**

This includes only those participants who strictly adhered to the protocol throughout the study. It excludes participants who had major protocol violations, didn't receive the full course of treatment, or didn't complete the study as planned.

﻿

**STATISTICAL ANALYSES**

Conducted by the trial's lead statistician, the primary analysis will undergo an independent review by a second statistician to ensure accuracy. The primary and secondary outcomes will primarily adhere to the ITT principle for the main analytical strategy.

**Primary Outcome Analysis**

Primary Outcome Assessment: The primary endpoint is categorized as binary, with a favorable functional outcome operationalized as a modified Rankin Scale (mRS) score within the range of 0-2 at 90 days post-treatment. Analysis of the primary outcome will utilize the ITT population as previously described. The endpoint will be presented as the count and percentage of participants achieving this excellent outcome, stratified by treatment group.

Statistical Analysis Approach: A formal statistical evaluation will employ log-binomial regression to analyze the likelihood of achieving the primary outcome. In this model, the binary occurrence of a favorable outcome at the 90-day mark serves as the dependent variable, with treatment group as the sole independent predictor. The analysis will yield the odds ratio for the primary outcome between the IA-LTH group and the Control group, accompanied by a two-sided 95% confidence interval (CI) and the associated p-value.

**Covariate adjusted analysis of the primary outcome**

Adjusted Analysis for Primary Endpoint: To ascertain the impact of covariates on the treatment effect, adjusted analyses will be conducted on the primary endpoint. The covariates to be incorporated in these adjusted analyses include:

- Age, stratified into two groups: aged ≥75 years and aged <75 years

- ASPECTS score at the time of randomization, stratified into two groups: ASPECTS ≥8 and ASPECTS <8

Utilizing the logistic regression model, the adjusted relative rate (RR), along with the 95% confidence interval (CI), will be calculated to compare the IA-LTH group with the Control group, accounting for these additional variables.

**Secondary Outcome**

Analysis Secondary outcome analyses will be based on the ITT populations.

**Analysis of binary outcomes**

Secondary Outcome Analysis: The percentage of participants achieving an mRS score of 0-1 at 90 days, experiencing death within the same period, and encountering early neurological deterioration within 24 hours are regarded as binary endpoints. These metrics will be tallied and presented as percentages of the total in each treatment cohort, mirroring the analytical approach for the primary outcome.

In the evaluation of these secondary endpoints, the relative risk (RR) along with its two-sided 95% confidence interval (CI) will be determined to contrast the IA-LTH group against the Control group. Utilizing log-binomial regression models, treatment will serve as the predictor variable, providing RR estimates that reflect the treatment's impact on these binary outcomes.

**Exploratory Analysis:**

Should the need arise, supplementary statistical techniques may be employed for a more in-depth exploration of the data. These methods are considered exploratory and will be applied judiciously to gain additional insights into the study's findings.

**GENERAL CONSIDERATIONS FOR DATA ANALYSES**

SPSS® (version 26) will be used to perform all data analyses.

**Covariates Analyses**

An examination of covariates will be conducted for both the primary and secondary outcomes within the ITT population. This analysis will evaluate the impact of various covariates on the outcomes. Additional covariate analyses may be undertaken based on the needs of the study and the presence of factors that may influence the results.

**Multiplicity**

The investigation of secondary outcomes, as well as supplementary analyses for the primary outcome, is considered exploratory. Consequently, adjustments for multiple comparisons will not be implemented in the evaluation of these outcomes.

**Missing data**

In the analysis adjusted for covariates, missing baseline covariate values will be addressed through straightforward imputation techniques that reflect the distribution of the covariates. Specifically, for continuous covariates, missing values will be substituted with the mean value derived from the existing sample data. In the case of categorical covariates, missing values will be replaced with the most prevalent category as determined by the sample.

**﻿Further Exploratory Analyses**

Additional exploratory analyses may be conducted at the discretion of the Trial Management Group (TMG) if deemed necessary. Such analyses will be incorporated into the analysis plan as an amendment, accompanied by appropriate justification.

1. Occurrence of mRS (0-1) at 90 days (binary outcome): The primary endpoint, indicative of an excellent functional status, is defined by the attainment of a modified Rankin Scale (mRS) score within the range of 0 to 1 at 90 days post-treatment.

2. Scores of mRS at 90 days (ordinal outcome): The distribution of mRS scores at 90 days provides an ordinal measure of functional outcomes, reflecting the spectrum of patient recovery.

3. Time from randomization to the occurrence of death of any cause at 90 days (time-to-event outcome): All-cause mortality is recorded from the time of randomization until death occurs within the 90-day study period.

4. Cerebral Hernia: The occurrence of brain herniation, a severe neurological complication, is closely monitored and recorded as part of safety assessments.

**Data Summaries**

Continuous data will be presented, detailing the count of valid observations (n), mean, standard deviation, median, and range. For continuous efficacy metrics, confidence intervals will be included. Categorical data will be tallied to show absolute frequencies and percentages per category. The percentage calculations will be based on the number of subjects in each treatment group with available data, unless otherwise indicated.

**References**

[1] Feigin, VL, Brainin, M, Norrving, B, et al., World Stroke Organization (WSO): Global Stroke Fact Sheet 2022, Int J Stroke, 2022;17:18-29.

[2] Virani, SS, Alonso, A, Aparicio, HJ, et al., Heart Disease and Stroke Statistics-2021 Update: A Report From the American Heart Association, Circulation, 2021;143:e254-e743.

[3] Huang, Y, Chen, S, Luo, Y, et al., Crosstalk between Inflammation and the BBB in Stroke, Curr Neuropharmacol, 2020;18:1227-1236.

[4] Piironen, K, Tiainen, M, Mustanoja, S, et al., Mild hypothermia after intravenous thrombolysis in patients with acute stroke: a randomized controlled trial, Stroke, 2014;45:486-491.

[5] McNamara, PJ, Jain, A, El-Khuffash, A, et al., Guidelines and Recommendations for Targeted Neonatal Echocardiography and Cardiac Point-of-Care Ultrasound in the Neonatal Intensive Care Unit: An Update from the American Society of Echocardiography, J Am Soc Echocardiogr, 2024;37:171-215.
