# Supplementary material for: Intra-arterial selective hypothermia for acute ischemic stroke neuroprotection: A multicenter pilot trial in China
Source: PLoS Med. 2025 Jul 24;22(7):e1004668. doi: 10.1371/journal.pmed.1004668 (PMC12289068; doi:10.1371/journal.pmed.1004668)
Supplement: S1 CONSORT Checklist — (DOCX) [file pmed.1004668.s005.docx]

|  | Section/topic | No | CONSORT 2025 checklist item description | Reported on page no. |
| --- | --- | --- | --- | --- |
|  | **Title and abstract** | | |  |
|  | Title and structured abstract | 1a | Identification as a randomised trial | Title: "A Multicenter Pilot Trial"; Methods, paragraph 1: "multicenter, randomized trial" |
|  |  | 1b | Structured summary of the trial design, methods, results, and conclusions | Abstract, all sections (Background, Methods and findings, Conclusions) |
|  | **Open science** | | | Abstract, Methods and findings; Methods, final paragraph: "ChiCTR2300074990 on August 22, 2023" |
|  | Trial registration | 2 | Name of trial registry, identifying number (with URL) and date of registration |  |
|  | Protocol and statistical analysis plan | 3 | Where the trial protocol and statistical analysis plan can be accessed | Methods, Trial Design, paragraph 1: "The protocol (S1-3 Appendix)... S4 Appendix" |
|  | Data sharing | 4 | Where and how the individual de-identified participant data (including data dictionary), statistical code and any other materials can be accessed | Data Availability Statement: "via email at [xuyingjie@mail.ustc.edu.cn](mailto:xuyingjie@mail.ustc.edu.cn)" |
|  | Funding and conflicts of interest | 5a | Sources of funding and other support (eg, supply of drugs), and role of funders in the design, conduct, analysis and reporting of the trial | Funding/Support section; "The funders had no role..." |
|  |  | 5b | Financial and other conflicts of interest of the manuscript authors | Conflict of Interest Disclosures: "None" |
|  | **Introduction** | | |  |
|  | Background and rationale | 6 | Scientific background and rationale | Introduction, paragraphs 1-7 |
|  | Objectives | 7 | Specific objectives related to benefits and harms | Introduction, final paragraph: "to test the hypothesis..." |
|  | **Methods** | | |  |
|  | Patient and public involvement | 8 | Details of patient or public involvement in the design, conduct and reporting of the trial | Not reported |
|  | Trial design | 9 | Description of trial design including type of trial (eg, parallel group, crossover), allocation ratio, and framework (eg, superiority, equivalence, non-inferiority, exploratory) | Methods, Trial Design, paragraph 1: "multicenter, prospective, open-label, randomized, blinded-endpoint trial" |
|  | Changes to trial protocol | 10 | Important changes to the trial after it commenced including any outcomes or analyses that were not prespecified, with reason | S3 Appendix_Summary of changes |
|  | Trial setting | 11 | Settings (eg, community, hospital) and locations (eg, countries, sites) where the trial was conducted | Methods, Trial Design: "18 centers in China" |
|  | Eligibility criteria | 12a | Eligibility criteria for participants | Methods, Participants section, paragraphs 1-3 |
|  |  | 12b | If applicable, eligibility criteria for sites and for individuals delivering the interventions (eg, surgeons, physiotherapists) | Methods, Procedures: "researchers received training on the trial protocol" |
|  | Intervention and comparator | 13 | Intervention and comparator with sufficient details to allow replication. If relevant, where additional materials describing the intervention and comparator (eg, intervention manual) can be accessed | Methods, Procedures section, paragraphs 1-3 |
|  | Outcomes | 14 | Prespecified primary and secondary outcomes, including the specific measurement variable (eg, systolic blood pressure), analysis metric (eg, change from baseline, final value, time to event), method of aggregation (eg, median, proportion), and time point for each outcome | Methods, Outcomes section, paragraphs 1-2 |
|  | Harms | 15 | How harms were defined and assessed (eg, systematically, non-systematically) | Methods, Outcomes: paragraphs 1-2  11.2.AEs and SAEs (S1-2 Appendix). |
|  | Sample size | 16a | How sample size was determined, including all assumptions supporting the sample size calculation | Methods, Statistical Analysis: "sample size of 100 cases was determined based on recommendations" |
|  |  | 16b | Explanation of any interim analyses and stopping guidelines | Not reported |
|  | Randomisation: |  |  |  |
|  | Sequence generation | 17a | Who generated the random allocation sequence and the method used | Methods, Randomization and Blinding: "independent statistician prepared the randomization list" |
|  |  | 17b | Type of randomisation and details of any restriction (eg, stratification, blocking and block size) | Methods, Randomization and Blinding: "stratified block randomization" with age and ASPECTS |
|  |  |  |  | **Reported on page no.** |
|  | Allocation concealment mechanism | 18 | Mechanism used to implement the random allocation sequence (eg, central computer/telephone; sequentially numbered, opaque, sealed containers), describing any steps to conceal the sequence until interventions were assigned | Methods, Randomization and Blinding: "central Interactive Web Response System (IWRS)" |
|  | Implementation | 19 | Whether the personnel who enrolled and those who assigned participants to the interventions had access to the random allocation sequence | Methods, Randomization and Blinding: "To maintain allocation concealment..." |
|  | Blinding | 20a | Who was blinded after assignment to interventions (eg, participants, care providers, outcome assessors, data analysts) | Methods, Randomization and Blinding: "outcome assessors and patients were blinded" |
|  |  | 20b | If blinded, how blinding was achieved and description of the similarity of interventions | Methods, Randomization and Blinding: "blinded evaluation procedure...blinded central adjudication committee" |
|  | Statistical methods | 21a | Statistical methods used to compare groups for primary and secondary outcomes, including harms | Methods, Statistical Analysis, paragraph 2 |
|  |  | 21b | Definition of who is included in each analysis (eg, all randomised participants), and in which group | Methods, Statistical Analysis: "intention-to-treat (ITT) approach" |
|  |  | 21c | How missing data were handled in the analysis | Methods, Statistical Analysis: "excluding those lost to follow-up or who withdrew consent" |
|  |  | 21d | Methods for any additional analyses (eg, subgroup and sensitivity analyses), distinguishing prespecified from post hoc | Methods, Statistical Analysis: "subgroup analysis was conducted" |
|  | **Results** | | |  |
|  | Participant flow, including flow diagram | 22a | For each group, the numbers of participants who were randomly assigned, received intended intervention, and were analysed for the primary outcome | Results, Trial population; Figure 1 |
|  |  | 22b | For each group, losses and exclusions after randomisation, together with reasons | Results, Trial population: "Two participants were subsequently excluded" |
|  | Recruitment | 23a | Dates defining the periods of recruitment and follow-up for outcomes of benefits and harms | Results, Trial population: "Between September 24, 2023, and January 10, 2024" |
|  |  | 23b | If relevant, why the trial ended or was stopped | Methods, final paragraph: "has now been closed at all participating sites" |
|  | Intervention and comparator delivery | 24a | Intervention and comparator as they were actually administered (eg, where appropriate, who delivered the intervention/comparator, how participants adhered, whether they were delivered as intended (fidelity)) | Results, implicitly throughout outcomes section |
|  |  | 24b | Concomitant care received during the trial for each group | Methods, Procedures: "standard medical care following national stroke guidelines" |
|  | Baseline data | 25 | A table showing baseline demographic and clinical characteristics for each group | Results, Table 1 |
|  | Numbers analysed,  outcomes and estimation | 26 | For each primary and secondary outcome, by group:  ● the number of participants included in the analysis  ● the number of participants with available data at the outcome time point  ● result for each group, and the estimated effect size and its precision (such as 95% confidence interval)  ● for binary outcomes, presentation of both absolute and relative effect size | Results, Primary and secondary outcomes; Table 2 |
|  | Harms | 27 | All harms or unintended events in each group | Results, Safety section; Table 3 |
|  | Ancillary analyses | 28 | Any other analyses performed, including subgroup and sensitivity analyses, distinguishing pre-specified from post hoc | Results, Primary and secondary outcomes: "subgroup analysis"; Figure 3 |
|  | **Discussion** | | |  |
|  | Interpretation | 29 | Interpretation consistent with results, balancing benefits and harms, and considering other relevant evidence | Discussion, paragraphs 1-3 |
|  | Limitations | 30 | Trial limitations, addressing sources of potential bias, imprecision, generalisability, and, if relevant, multiplicity of analyses | Abstract, Methods and findings; Discussion, paragraph 4 |

Citation: Hopewell S, Chan AW, Collins GS, Hróbjartsson A, Moher D, Schulz KF, et al. CONSORT 2025 Statement: updated guideline for reporting randomised trials. BMJ. 2025; 388:e081123. <https://dx.doi.org/10.1136/bmj-2024-081123>
© 2025 Hopewell et al. This is an Open Access article distributed under the terms of the Creative Commons Attribution License (<https://creativecommons.org/licenses/by/4.0/>), which permits unrestricted use, distribution, and reproduction in any medium, provided the original work is properly cited.

*We strongly recommend reading this statement in conjunction with the CONSORT 2025 Explanation and Elaboration and/or the CONSORT 2025 Expanded Checklist for important clarifications on all the items. We also recommend reading relevant CONSORT extensions. See [www.consort-spirit.org](http://www.consort-spirit.org).
