# Supplementary material for: Intra-arterial selective hypothermia for acute ischemic stroke neuroprotection: A multicenter pilot trial in China
Source: PLoS Med. 2025 Jul 24;22(7):e1004668. doi: 10.1371/journal.pmed.1004668 (PMC12289068; doi:10.1371/journal.pmed.1004668)
Supplement: S1 Graphical Abstract — (PDF) [file pmed.1004668.s007.pdf]

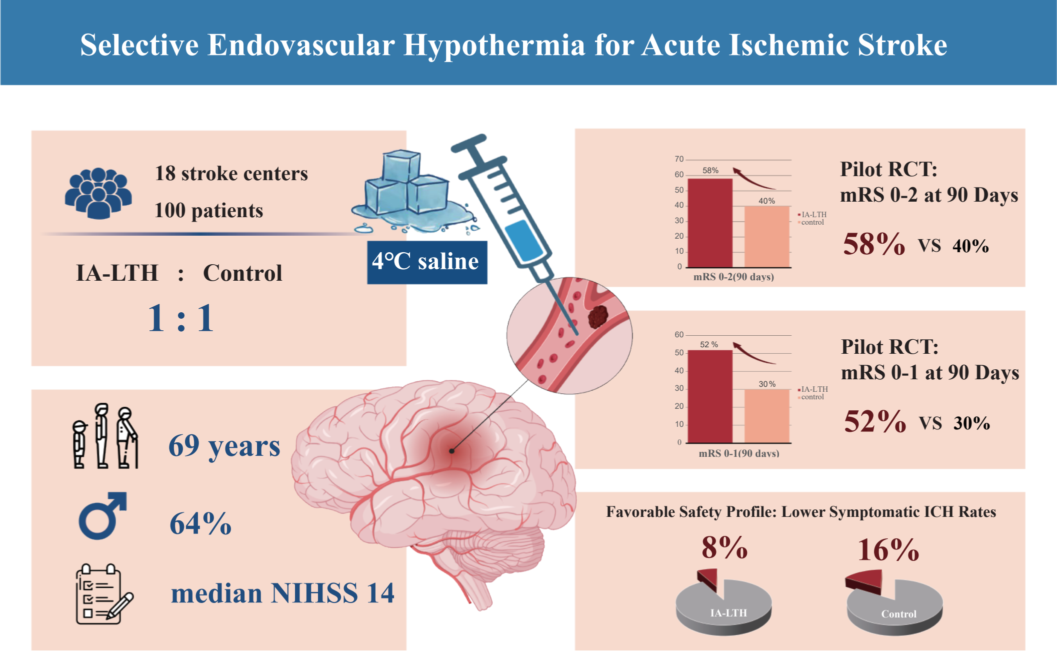

Created in BioRender. Tu, D. (2025) https://BioRender.com/hg62nnc
